# CHARGE syndrome protein CHD7 regulates epigenomic activation of enhancers in granule cell precursors and gyrification of the cerebellum

**DOI:** 10.1038/s41467-021-25846-3

**Published:** 2021-09-29

**Authors:** Naveen C. Reddy, Shahriyar P. Majidi, Lingchun Kong, Mati Nemera, Cole J. Ferguson, Michael Moore, Tassia M. Goncalves, Hai-Kun Liu, James A. J. Fitzpatrick, Guoyan Zhao, Tomoko Yamada, Azad Bonni, Harrison W. Gabel

**Affiliations:** 1 Department of Neuroscience, Washington University School of Medicine, St. Louis, MO 63110 USA; 2 MD-PhD Program, Washington University School of Medicine, St. Louis, MO 63110 USA; 3 Division of Molecular Neurogenetics, DKFZ-ZMBH Alliance, German Cancer Research Center Im Neunheimer Feld 280, 69120 Heidelberg, Germany; 4 Department of Cell Biology & Physiology, Washington University School of Medicine, St. Louis, MO 63110 USA; 5 Department of Biomedical Engineering, Washington University in St. Louis, St. Louis, MO 63130 USA; 6 Washington University Center for Cellular Imaging, Washington University School of Medicine, St. Louis, MO 63110 USA; 7 Department of Neurobiology, Northwestern University, Evanston, IL 60201 USA

**Keywords:** Morphogenesis, Gene expression, Epigenetics, Development of the nervous system, Epigenetics in the nervous system

## Abstract

Regulation of chromatin plays fundamental roles in the development of the brain. Haploinsufficiency of the chromatin remodeling enzyme CHD7 causes CHARGE syndrome, a genetic disorder that affects the development of the cerebellum. However, how CHD7 controls chromatin states in the cerebellum remains incompletely understood. Using conditional knockout of CHD7 in granule cell precursors in the mouse cerebellum, we find that CHD7 robustly promotes chromatin accessibility, active histone modifications, and RNA polymerase recruitment at enhancers. In vivo profiling of genome architecture reveals that CHD7 concordantly regulates epigenomic modifications associated with enhancer activation and gene expression of topologically-interacting genes. Genome and gene ontology studies show that CHD7-regulated enhancers are associated with genes that control brain tissue morphogenesis. Accordingly, conditional knockout of CHD7 triggers a striking phenotype of cerebellar polymicrogyria, which we have also found in a case of CHARGE syndrome. Finally, we uncover a CHD7-dependent switch in the preferred orientation of granule cell precursor division in the developing cerebellum, providing a potential cellular basis for the cerebellar polymicrogyria phenotype upon loss of CHD7. Collectively, our findings define epigenomic regulation by CHD7 in granule cell precursors and identify abnormal cerebellar patterning upon CHD7 depletion, with potential implications for our understanding of CHARGE syndrome.

## Abstract (web summary)

CHARGE syndrome that affects cerebellar development can be caused by haploinsufficiency of the chromatin remodeling enzyme CHD7; however the precise role of CHD7 remains unknown. Here the authors show CHD7 promotes chromatin accessibility and enhancer activity in granule cell precursors and regulates morphogenesis of the cerebellar cortex, where loss of CHD7 triggers cerebellar polymicrogyria.

## Introduction

Remodeling of chromatin represents a fundamental epigenetic mechanism of transcriptional control^1,2^. Among enzymes that remodel chromatin, the chromodomain helicase DNA-binding (CHD) family of ATP-dependent chromatin remodelers plays critical roles in brain development^3–5^. Accordingly, mutations of several CHD family proteins have been implicated in neurodevelopmental disorders of cognition^6–9^. Prominent among these mutations, haploinsufficiency of the chromatin remodeling enzyme CHD7 causes CHARGE syndrome, a multisystem disorder that includes a wide range of neurodevelopmental defects^10,11^. However, how CHD7 controls chromatin states in the developing brain remains incompletely understood.

Within the brain, hypoplasia of the cerebellum appears to be a major morphological abnormality in CHARGE syndrome^12–14^. Consistent with this observation, CHD7 is highly expressed in granule cells of the cerebellum^15,16^. Thus, understanding CHD7 function in the regulation of the epigenome in granule cells of the cerebellum may advance our understanding of both chromatin remodeling in the brain as well as the pathogenesis of CHARGE syndrome.

Studies of CHD7 function in the mouse cerebellum have shown that CHD7 controls chromatin accessibility at distal regulatory elements in the cerebellum^15,16^. As enhancers are critical regulators of gene expression, elucidating the role of CHD7 at enhancers will be critical to understanding how CHD7 controls the epigenome and gene expression. Recent studies have revealed that the chromatin remodeling protein CHD4 regulates genome architectural changes at enhancers in the brain^17^. However, the extent to which other chromatin remodeling enzymes including CHD7 influence genome architecture remains unknown. In addition, whether gene expression correlates with epigenetic alterations induced by CHD7 at topologically associated enhancers remains to be explored. Finally, CHD7 chromatin remodeling function remains to be studied in the context of a multilayered epigenetic regulatory program that integrates genome architecture with chromatin accessibility, transcriptional activity state, and gene expression.

CHD proteins may control developmental events in the brain in a spatiotemporal-specific manner^4,5^. In the mouse cerebellum, CHD7 activity spans a significant extent of granule cell development, beginning with generation of granule cell precursors between embryonic day 12.5 and 17 (E12.5 and E17)^16^. CHD7 expression continues as granule cell precursors migrate from the rhombic lip to the external granule layer (EGL) within the developing cerebellum^16^. The expression of CHD7 persists in granule cell precursors in the postnatal mouse cerebellum when these cells rapidly proliferate within the EGL^16^. The dramatic expansion of granule cell precursors in the EGL provides the pool of cells that differentiate into granule neurons, representing the most numerous neurons in the brain, which migrate to the internal granule layer (IGL) within the cerebellar cortex^18–20^. The rapid expansion of granule cell precursors in the EGL is also thought to contribute to the morphogenesis of the cerebellar cortex, inducing its foliation in a stereotypical lobulation pattern along the anterior–posterior axis^21,22^. Recent studies identifying cerebellar hypoplasia upon CHD7 depletion suggest that CHD7 loss affects cerebellar cortical morphogenesis^15,16,23^. We, therefore, performed a high-resolution assessment of cerebellar patterning following loss of CHD7.

In this study, we define CHD7 function in the regulation of the epigenome in granule cell precursors and characterize a role for CHD7 in cerebellar cortical morphogenesis. Using conditional knockout of CHD7 in granule cell precursors of the mouse cerebellum, we find that CHD7 concomitantly promotes chromatin accessibility, active histone modifications, and RNA polymerase recruitment at enhancers. Remarkably, genome architecture analyses of the cerebellum in control and conditional CHD7 knockout mice reveal that CHD7-dependent regulation of local chromatin correlates with changes in the expression of genes within the same topologically associated domain (TAD) as CHD7-regulated enhancers. Notably, CHD7-regulated enhancers are associated with genes that control brain tissue morphogenesis. Strikingly, conditional knockout of CHD7 triggers pronounced and fully penetrant formation of polymicrogyria along the normally smooth mediolateral axis of the cerebellar vermis. At a cellular level, we uncover that conditional CHD7 knockout leads to a switch in the preferred axis of granule cell precursor division. Consistent with these findings in mice, genes bound by CHD7 in the fetal human cerebellum are implicated in disorders of brain folding. We also identify aberrant cerebellar folding in the brain of a patient with CHARGE syndrome. Collectively, our findings suggest that CHD7 governs enhancer and transcriptional activation in granule cell precursors during a window of cerebellar development that controls cerebellar cortical morphogenesis, thereby offering potential mechanisms by which epigenetic factors contribute to brain folding during development and disease.

## Results

### CHD7 occupies accessible enhancers characterized by temporally distinct epigenomic states

To elucidate the epigenetic regulatory role of CHD7 during cerebellar development, we first characterized the genomic binding of CHD7 in the mouse cerebellum at postnatal day 4 (P4), a period of peak granule cell precursor expansion. To determine the specificity of CHD7 genomic binding, we employed an Atoh1 (Math1) enhancer-driven Cre transgenic line to conditionally trigger knockout of the *Chd7* gene selectively in the granule cell lineage at the time of specification in the rhombic lip^15^. Immunohistochemical analyses revealed CHD7 depletion in granule cell precursors in the anterior and central cerebellar vermis upon conditional knockout of CHD7 using the Math1-Cre line (Supplementary Fig. 1a, b)^15,16^. Immunoblotting analyses of micro-dissected anterior cerebellar vermis showed effective depletion of CHD7 in conditional CHD7 knockout mice (Supplementary Fig. 1c).

**Fig. 1:**
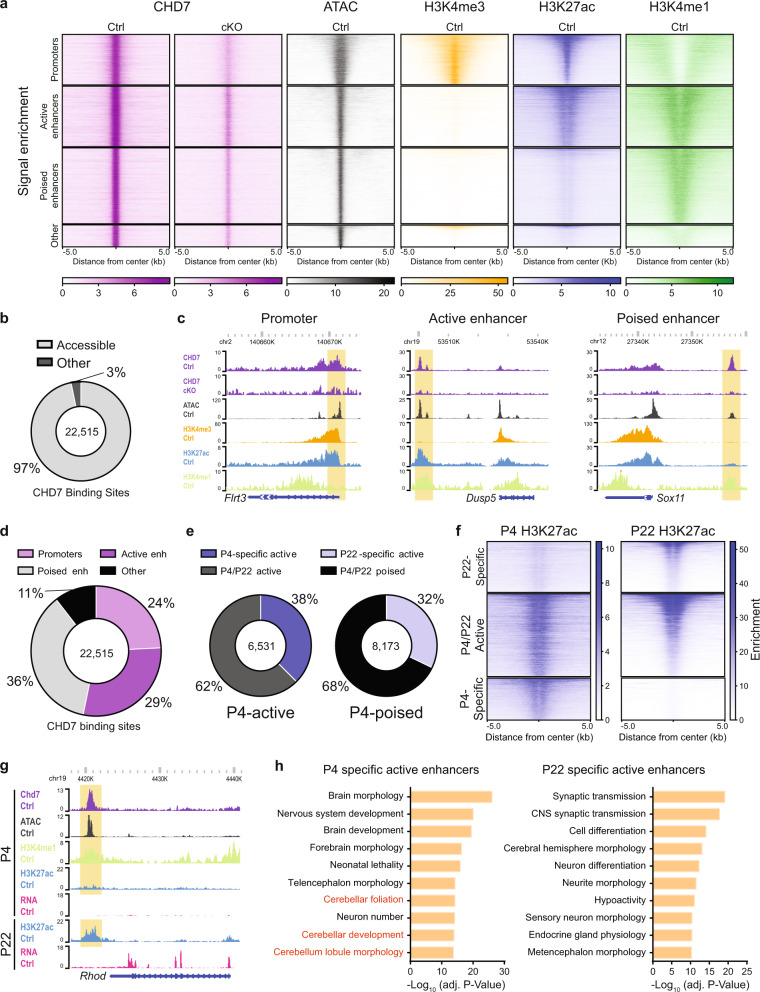
CHD7 occupies accessible enhancers characterized by temporally distinct activity states. **a** Heatmap of ChIP-seq signal for CHD7 (purple), H3K4me3 (orange), H3K27ac (blue), H3K4me1 (green) and ATAC-seq (black) centered on CHD7 genomic binding sites (*n* = 22,515). Heatmaps are split into four groups (promoters, active enhancers, poised enhancers, and other). Anterior cerebellum from P4 mice were used (*n* = 2 for CHD7 Ctrl, *n* = 1 for CHD7 cKO, *n* = 3 for histone modifications and ATAC-seq). **b** Pie chart depicting the proportion of CHD7-binding sites found to overlap ATAC-seq peaks (accessible regions) and not overlap (other). **c** WashU Epigenome Browser view of a promoter (left), active enhancer (middle), and poised enhancer (right) region (highlighted). Each site shows ChIP-seq coverage of CHD7, H3K4me3, H3K27ac, H3K4me1, and ATAC-seq. **d** Pie chart displaying regulatory element distribution of CHD7 peaks. Enh, enhancer. **e** Pie charts displaying the distribution of CHD7-bound P4-active (left) and P4-poised (right) enhancers at P4 (*n* = 3) and P22 (*n* = 3). **f** Heatmap of P4 and P22 H3K27ac ChIP-seq (*n* = 3 for each timepoint) centered on CHD7 developmentally active enhancer binding sites (*n* = 14,704). Heatmaps are split into three groups (P4-specific, P4/P22-active, and P4specific). **g** WashU Epigenome Browser view of a CHD7-bound P4 poised, P22-active enhancer (highlighted). ChIP-seq coverage for CHD7, H3K27ac, H3K4me1, as well as, ATAC-seq and RNA-seq is shown. **h** GREAT analysis showing enriched mouse phenotypes associated with P4-specific active enhancers (left) and P22-specific active enhancers (right).

Chromatin immunoprecipitation followed by sequencing (ChIP-seq) in the anterior cerebellum from P4 mice using an antibody to CHD7 showed that CHD7-occupied 22,515 genomic sites. CHD7 ChIP signal was reduced in conditional CHD7 knockout mice, validating the specificity of CHD7 ChIP signal (Fig. 1a). Intersection of these analyses with an assay for transposase-accessible chromatin followed by sequencing (ATAC-seq) revealed that CHD7 predominantly occupied accessible genomic regions (Fig. 1a, b). Systematic ChIP-seq analyses of histone modifications identified most CHD7-bound regions as promoters displaying strong H3K4me3 signal or enhancers displaying H3K4me1 signal (Fig. 1a)^24^. Based on the presence or absence of the histone modification H3K27 acetylation (H3K27ac), following the conventions of the field^25^, we further classified CHD7-bound enhancers as active or poised (Fig. 1a, c). For example, CHD7 bound an active enhancer of *Dusp5*, marked by H3K4me1 and H3K27ac (Fig. 1c). By contrast, CHD7 bound a poised enhancer upstream of *Sox11*, characterized by an H3K4me1-positive, H3K27ac-negative region (Fig. 1c). In sum, CHD7-bound regulatory elements were composed of 24.2% promoters, 29.0% active enhancers, and 36.3% poised enhancers (Fig. 1d). These results indicate that CHD7 occupies diverse accessible genomic regulatory elements in granule cell precursors of the developing cerebellum.

As poised enhancers may have the potential for developmental regulation^26,27^, we next characterized the developmental profile of CHD7-occupied poised enhancer sites. Comparison of H3K27ac levels at P4 and P22 at these CHD7-occupied poised enhancer sites showed that 32.2% of poised enhancers in the cerebellum in P4 mice became significantly active at P22 (deemed P22-specific active enhancers). Notably, 62.5% of enhancers active at P4 remained active at P22 and were designated as P4/P22-active. However, 37.5% of P4-active enhancers displayed significantly reduced H3K27ac levels at P22, and thus were termed P4-specific active enhancers (Fig. 1e, f). An illustrative example of a P22-specific active enhancer was found upstream of the *RhoD* gene, which at P4 showed H3K4me1 signal and minimal H3K27ac signal, and by P22 the enhancer showed H3K4me1 and H3K27ac signal (Fig. 1g). Correspondingly, transcription of *RhoD* was minimal at P4 and became significantly elevated by P22 (Fig. 1g). By contrast, an enhancer within the *Cdk6* gene was H3K27ac-positive at P4, and had reduced H3K27ac levels at P22 (Supplementary Fig. 1d), providing an example of a P4-specific active enhancer. Consistently, transcription of the *Cdk6* gene was evident at P4 but was significantly reduced at P22 (Supplementary Fig. 1d).

We next characterized the genomic context of CHD7-occupied enhancers from each developmental profile. Gene ontology analyses revealed that CHD7-occupied P4-specific active enhancers were associated with genes, such as *Neurod1*, *Ptch1*, and *Lama1*, encoding proteins implicated in cerebellar tissue morphogenesis. By contrast, P22-specific active enhancers were associated with genes, such as *Cacna1c*, *Grin2a*, and *Pclo*, encoding proteins that control synaptic and neuronal differentiation (Fig. 1h and Supplementary Data 1). Interestingly, enhancers active at both P4 and P22 were associated with genes, such as *Synpo*, *Slc6a1*, and *Neurl1a*, encoding proteins important for higher order processes such as learning, cognition, and locomotor coordination (Supplementary Fig. 1e and Supplementary Data 1). Additionally, CHD7-negative active enhancers are associated with some but not all of the gene classes as CHD7-occupied active enhancers (Supplementary Fig. 1f and Supplementary Data 1). Collectively, these data suggest that CHD7 may operate at distinct enhancers in a temporally specific manner during brain development.

### CHD7 regulates chromatin accessibility and epigenomic state of enhancers

**Fig. 2:**
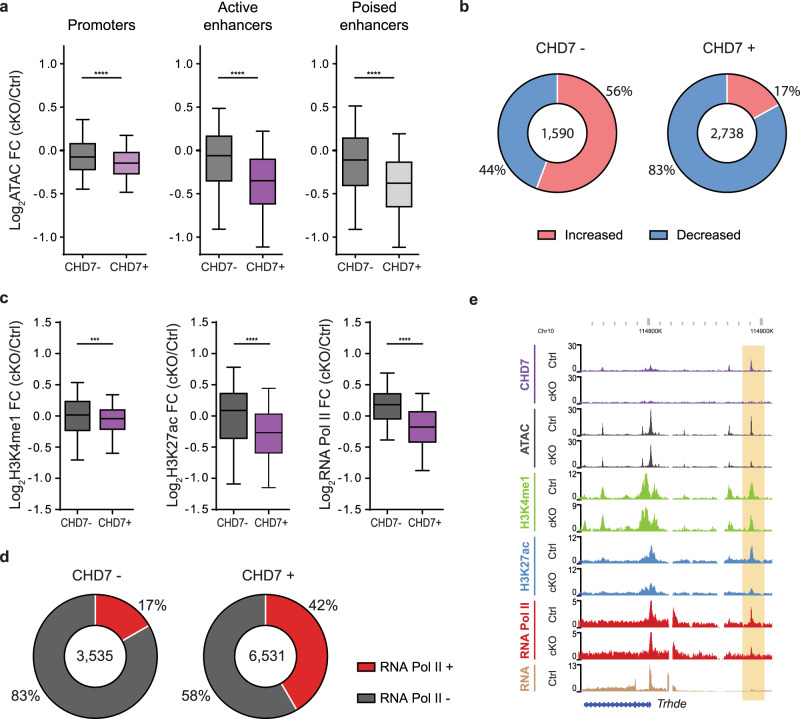
CHD7 regulates chromatin accessibility and activity state at enhancers. **a** Box-whisker plots showing the median, 1st quartile, 3rd quartile, and whiskers displaying the 5th and 95th percentiles of promoters (*n* = 11,398, *n* = 5238), active enhancers (*n* = 3535, *n* = 6531), and poised enhancers (*n* = 25,231, *n* = 8039) representing median and distribution of the log2 fold-change of ATAC-seq signal in CHD7 cKO over CHD7 control. CHD7 unoccupied, (CHD7–) left, and occupied, (CHD7+) right, enhancers are shown. ****p < 10^−4^, unpaired two-sided *t*-test, Welch’s correction. **b** Pie chart representing proportion active enhancers that show significantly changed accessibility in the CHD7 cKO, which display either increased or reduced accessibility. **c** Box-whisker plots showing the median, 1st quartile, 3rd quartile, and whiskers displaying the 5th and 95th percentiles of active enhancers representing median and distribution of the log2 fold-change of H3K4me1 (left; *n* = 760, *n* = 2457), H3K27ac (middle; *n* = 1590, *n* = 2738), and RNApolII (right; *n* = 249, *n* = 1099) signal in CHD7 cKO over CHD7 control. ***p < 10^–3^, ****p < 10^− 4^, unpaired two-sided *t*-test, Welch’s correction. **d** Pie chart representing proportion of CHD7 unoccupied (CHD7−; left) and occupied (CHD7+; right) active enhancers displaying RNApolII binding. **e** WashU Epigenome Browser view of a CHD7-bound active enhancer (highlighted) near the *Trhde* gene. ChIP-seq coverage for CHD7, H3K27ac, H3K4me1, RNApolII, as well as ATAC-seq and RNA-seq is shown.

To characterize the nucleosome remodeling activity of CHD7 in the brain, we assessed the effect of conditional knockout of CHD7 on chromatin accessibility in granule cell precursors. In ATAC-seq analyses of the anterior cerebellar vermis in P4 mice, depletion of CHD7 significantly reduced chromatin accessibility at CHD7-bound sites relative to non-bound regulatory elements (Fig. 2a). Strikingly, 83% of CHD7-bound active enhancers exhibited decreased chromatin accessibility upon CHD7 depletion, whereas differential accessibility at non-bound active enhancers showed minimal directional preference (Fig. 2b). These results suggest that CHD7 predominantly promotes chromatin accessibility at enhancers in granule cell precursors.

We next characterized whether regulation of chromatin accessibility by CHD7 might correlate with epigenomic modifications associated with enhancer activation in granule cell precursors in the anterior cerebellar vermis of P4 mice. Enhancers undergo a series of modifications during activation, beginning with the generation of nucleosome free regions by pioneer transcription factors and chromatin remodeling complexes^28,29^, the pre-marking with H3K4me1of enhancers poised for activation^25,27^, and the subsequent acquisition of H3K27ac, a mark associated with active enhancers, and recruitment of RNA polymerase II (RNAPII)^25,30^. Therefore, we assessed the effect of CHD7 conditional knockout on enhancer levels of H3K4me1, H3K27ac and RNAPII. Analysis of the poised mark H3K4me1 showed limited changes in signal at CHD7-bound sites in granule cell precursors upon conditional CHD7 knockout. By contrast, H3K27ac ChIP-seq signal at CHD7-bound sites was significantly reduced. Moreover, conditional CHD7 knockout also reduced the binding of RNAPII at CHD7-bound active enhancers in granule cell precursors (Fig. 2c). Notably, RNAPII was present at 42% of CHD7-bound active enhancers (Fig. 2d). By contrast, RNAPII was found at only 17% at non-bound active enhancers in granule cell precursors, suggesting that CHD7 may trigger enrichment and stabilization of RNAPII at active enhancers (Fig. 2d and Supplementary Fig. 1g). By way of an example, conditional CHD7 knockout reduced chromatin accessibility, H3K27ac levels, and RNAPII binding but not H3K4me1 levels, at the active *Trhde* gene enhancer in granule cell precursors (Fig. 2e). Thus, these findings suggest that CHD7 primarily influences epigenomic marks associated with enhancer activation, with relatively minimal effect on a significant mark of enhancer poising. Together, our data support a model in which CHD7 concomitantly promotes chromatin accessibility and acetylation at enhancers to regulate transcriptional programs in the brain.

### CHD7 chromatin remodeling at enhancers corresponds to transcriptional activity at topologically associated promoters

We next assessed how epigenomic alteration of enhancers following depletion of CHD7 is associated with changes in gene expression. The activity of the chromatin remodeler CHD4 influences genome architecture and enhancer activation to regulate developmental gene expression in the cerebellum^17^. We asked if CHD7 might influence gene expression via regulation of chromatin conformation. First, we characterized genome architecture in the anterior cerebellar vermis of P4 conditional CHD7 knockout and sex-matched control littermates using in situ chromosome conformation capture with high-throughput sequencing (Hi-C) and ChIP-seq of the chromatin architecture proteins CTCF and RAD21. We combined these analyses with RNA-sequencing (RNA-seq) in the anterior cerebellar vermis in P4 mice to determine in an integrative manner how loss of CHD7 affects global genome architecture, enhancer–promoter contacts, and gene expression in the brain.

**Fig. 3:**
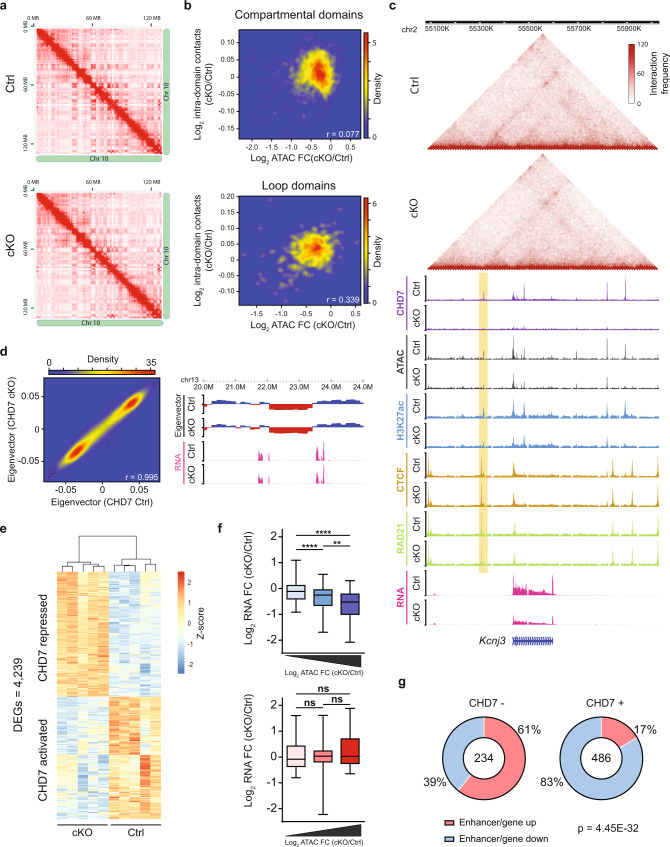
CHD7 chromatin remodeling at enhancers corresponds to transcriptional activity at topologically associated promoters. **a** Hi-C contact matrices of chromosome 10 in CHD7 control (top) and CHD7 cKO (bottom) cerebellum, visualized at chromosome-scale (*n* = 3 for each condition). Pixel intensity represents the normalized number of contacts between a pair of loci. Contact frequencies calculated at 10 Kb. **b** Density plots comparing the log2 fold-change in ATAC-seq signal in each contact domain to the log2 fold-change in Hi-C contacts for that domain. Compartmental domains (top) and loop domains (bottom). **c** WashU Epigenome Browser view of a loop domain. ChIP-seq coverage for CHD7, H3K27ac, Rad21, CTCF, as well as ATAC-seq and Hi-C, in CHD7 control and CHD7 cKO are shown. **d** Density plot comparing the eigenvectors calculated for 150 Kb bins in CHD7 control and cKO cerebellum (left). WashU Epigenome Browser view of eigenvectors depicting A/B compartments and RNA levels in CHD7 control and cKO cerebellum (right). **e** Hierarchical clustering of gene expression genes detected as significantly dysregulated (false-discovery rate [FDR],<0.05) in analysis of RNA-seq from micro-dissected Ctrl and cKO P4 anterior cerebellum (*n* = 5 biological replicates per genotype). Color represents *Z*-score of log2 cpm (counts per million) for a given gene. **f** Box-whisker plots showing the median, 1st quartile, 3rd quartile, and whiskers displaying the 5th and 95th percentiles of log2 fold-change of RNA signal for genes proximal to CHD7-bound sites with significant changes in accessibility in the CHD7 cKO. Active enhancers were divided into three groups based on significantly increasing or decreasing changes in ATAC-seq signal. We then quantified the change in gene expression of corresponding genes within groups defined by ATAC-seq changes. Genes must be found within 100 Kb and within the same TAD as the altered accessibility site to be linked to the site. Boxplots are divided into groups of decreasing (top; *n* = 255, *n* = 185, *n* = 129) and increasing (bottom; *n* = 39, *n* = 23, *n* = 22) ATAC-seq fold-change. ****p < 10^−4^, unpaired two-sided *t*-test, Tukey’s multiple comparison test. **g** Pie chart representing proportion of significantly changed enhancers in the CHD7 cKO for which transcript levels of the gene linked to that enhancer by an E–P loop change in the same direction as the enhancer. CHD7-occupied (CHD7+; right) enhancers are significantly different from unoccupied enhancers (CHD7–; left). *p*-value = 4.45E-32, one-sided Chi-squared test.

Hi-C data analyses from three biological replicates of conditional CHD7 knockout and control mice identified over 1.6 billion genomic contacts per genotype at 10 kilobase (kb) resolution (Fig. 3a and Supplementary Fig. 2a). Hi-C reproducibility analysis showed strong consistency among biological replicates, allowing us to pool data for downstream analyses, thereby effectively increasing sequencing depth and the ability to identify genomic contacts (Supplementary Fig. 2b).

We first assessed the role of CHD7 in organizing TADs. Using the Arrowhead algorithm, we identified 2373 TADs in the cerebellum of conditional CHD7 knockout and control mice (Supplementary Fig. 2c). Further analysis using HiCCUPS algorithm revealed 13,559 loops (Supplementary Fig. 2c). Among TAD domains, 602 TADs harbored genomic loops at domain borders, thus classified as loop domains, and 1,771 TADs did not contain border loops, and were thus classified as compartmental domains (Supplementary Fig. 2c).

Surprisingly, although conditional CHD7 knockout in granule cell precursors led to significant epigenomic changes at enhancers within contact domains, intradomain loops appeared largely unaltered in conditional CHD7 knockout mice compared to control littermates (Supplementary Fig. 2d). Within compartmental domains, the frequency of genomic interaction correlated poorly with changes in accessibility and H3K27ac levels at enhancers (Fig. 3b and Supplementary Fig. 2e). A similar analysis for loop domains revealed a modest correlation of genomic interaction frequency with enhancer accessibility and H3K27ac levels (Fig. 3b and Supplementary Fig. 2e). Correspondingly, ChIP-seq analyses of genome architectural proteins RAD21, a subunit of the Cohesin complex, and CTCF showed minimal changes in genome-wide occupancy in the anterior cerebellar vermis upon conditional knockout of CHD7 (Supplementary Fig. 2f, g). For example, genomic interactions within a loop domain near the gene *Kcnj3* were minimally altered upon loss of CHD7 in tandem with unaltered binding of RAD21 and CTCF (Fig. 3c). Furthermore, compartmentalization analyses revealed little change in A/B compartments and a high eigenvector correlation between conditional CHD7 knockout and control cerebellum (Fig. 3d). Collectively, these data suggest that CHD7 chromatin remodeling activity at enhancers occurs independently of alterations in genome architecture.

We next assessed if alterations in epigenomic states at enhancers in conditional CHD7 knockout mice corresponds to differential expression of genes that remain linked to these enhancers by stable genomic contacts. In RNA-seq analysis of the anterior cerebellum of P4 conditional CHD7 knockout and sex-matched control littermate mice, 2144 genes were significantly upregulated and 2095 genes were significantly downregulated upon conditional CHD7 knockout (Fig. 3e and Supplementary Fig. 3a). Through intersection of RNA-seq and TAD genomic architecture characterization, we found that CHD7-bound enhancers within a TAD displayed a Pearson correlation coefficient of 0.346 when comparing changes in accessibility to gene expression levels by RNA-seq (Supplementary Fig. 3b). Additionally, further analyses showed a concordant reduction in accessibility and the expression of topologically associated genes (Fig. 3f and Supplementary Fig. 3c). By contrast, the minor population of CHD7-bound enhancers with increased accessibility in conditional CHD7 knockout mice were not associated with upregulated expression of genes within the same TAD (Fig. 3f).

We further characterized enhancer–promoter (E–P) interactions and their relationship to CHD7 regulation of enhancers by assessing discrete loops in the Hi-C data linking enhancers and promoters. This analysis identified a total of 5175 E–P interactions that also feature loops in the anterior cerebellum in control and conditional CHD7 knockout mice. Despite significant epigenomic changes, conditional CHD7 knockout had little or no effect on E–P interactions (Supplementary Fig. 3d). Notably, CHD7 was preferentially bound to active enhancers within E–P interactions that displayed concordant decreases in enhancer accessibility and gene expression upon CHD7 depletion (Fig. 3g). In contrast, active enhancers within E–P interactions that did not bind CHD7 did not display a strong preference in either direction in changes of accessibility and gene expression (Fig. 3g). For E–P interactions containing enhancers that were significantly altered in conditional CHD7 knockout mice, we noted a Pearson correlation coefficient of 0.358 when comparing changes in domain accessibility and gene expression levels by RNA-seq (Supplementary Fig. 3e). Additionally, at enhancers that were significantly downregulated in conditional CHD7 knockout mice, gene expression was significantly reduced with decreasing enhancer accessibility, whereas upregulated genes showed no trend with increasing enhancer accessibility (Supplementary Fig. 3f). Altogether, these findings suggest that loss of CHD7 alters local chromatin structure at enhancers and leads to changes in the expression of genes that are linked by enhancer–promoter contacts.

To understand the potential consequences resulting from loss of CHD7, we next identified enriched CHD7-activated and CHD7-repressed biological processes by performing gene ontology analysis of significantly downregulated and upregulated genes, respectively. CHD7-activated biological processes were strongly enriched for genes necessary for a variety of neuronal developmental processes, such as neurogenesis and neuron differentiation. Notably, a cluster of CHD7-activated biological processes was enriched for genes required for the repression of gene expression, including terms such as, repression of gene expression, epigenetic and chromatin organization, transcriptional repression (Supplementary Data 1) Thus, while CHD7 appears to directly promote accessibility at regulatory elements, CHD7 may concordantly activate a subset of transcriptionally repressive gene pathways. Transcriptionally repressive downstream effectors of CHD7 might potentially underlie the significant upregulation of genes observed upon conditional CHD7 knockout. However, in view of the multiple functional domains of CHD7, we cannot exclude that CHD7 may directly repress regulatory elements in certain contexts. Our analysis of upregulated genes identified that most CHD7-repressed biological processes were enriched for genes necessary for proper extracellular signaling processes, including extracellular structure organization, cell surface receptor signaling, and cell communication (Supplementary Fig. 3a and Supplementary Data 1). Altogether, these data suggest that CHD7 concordantly regulates the epigenome at enhancers associated with CHD7-activated genes, while potentially reducing the expression of other genes via the activation of transcriptionally repressive downstream effectors.

### Conditional knockout of CHD7 results in stereotyped cerebellar microgyria

**Fig. 4:**
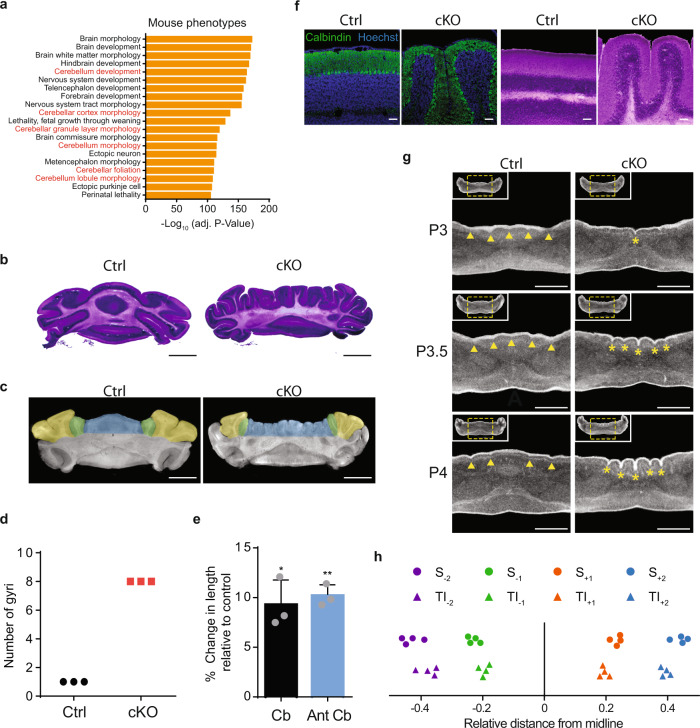
Conditional knockout of CHD7 results in stereotyped cerebellar microgyria. **a** GREAT analysis showing enriched mouse phenotypes associated with CHD7-regulated genes. Cerebellar phenotypes shown in red text. **b** Hematoxylin and eosin staining of Ctrl (left) and CHD7 cKO (right) p56 sections of mouse cerebellum. Scale bar: 2 mm. **c** Zeiss Xradia Versa 520 XRM was used to perform a nano-computerized tomography (nano CT) scan of Ctrl (left) and cKO (right) p56 mouse cerebella. The cerebellar vermis is highlighted blue and corresponding hemisphere regions highlighted green and yellow. Scale bar: 2 mm. **d** Graph showing number of gyri in the cerebellar vermis of the anterior basal lobe of Ctrl and cKO mice (*n* = 3 for each condition). **e** Bar graphs showing % change in total cerebellar (Cb) and anterior lobules (Ant Cb) lengths upon CHD7 cKO (*n* = 3 for each condition). Length is shown as mean ± s.e.m for each measurement. *p* = 0.0188 (Cb), *p* = 0.0024 (Ant Cb) Paired two-sided *t*-test. **f** IHC (left) and H&E (right) of Ctrl and cKO p56 mouse cerebellar sections. Experiment was repeated three times for each condition. Scale bars: 100 µm. **g** Nano CT scans were taken of P3, P3.5, and P4 mice cerebella. Digital 2D sections were taken through anterior basal lobe lobules. Insets show entire cerebellar section. Yellow dashed squares define region of focus. Yellow arrowheads on P4 Ctrl section mark transient vermal indentations. Yellow asterisks on P4 cKO section mark aberrant sulci. Scale bars: 500 µm. **h** Dot plot showing relative distance of transient invaginations (TI) in P4 CHD7 ctrl mice (*n* = 4) and sulci (S) formation in P4 CHD7 cKO mice (*n* = 4).

Having characterized the chromatin remodeling function of CHD7 in granule cell precursors, we next sought to investigate the biological consequences of CHD7 depletion in the developing cerebellum. Mouse phenotype analyses of significantly dysregulated genes suggested that CHD7 regulates genes, such as *Pax6*, *En1*, and *Neurod1*, that may play a role in cerebellar morphogenesis (Fig. 4a and Supplementary Data 1). Therefore, we characterized the effects of CHD7 depletion on cerebellar tissue morphogenesis. Remarkably, we observed a striking pattern of ectopic cerebellar folds along the normally smooth mediolateral axis of the anterior adult cerebellum, which we refer to as cerebellar microgyria (Fig. 4b). Histological analyses and nano-computerized tomography (CT) scan of the adult cerebellum in conditional CHD7 knockout mice revealed that the spatial organization of cerebellar microgyria was highly consistent between mice (Fig. 4c). In stark contrast to the normally smooth cerebellar surface in control mice, eight folds were detected along the mediolateral axis of the anterior cerebellar vermis in adult conditional CHD7 knockout mice with 100% penetrance (Fig. 4c, d). However, the posterior regions of the cerebellum that retain CHD7 expression in the conditional knockout displayed their normally smooth cerebellar surface (Supplementary Fig. 4a). In contrast to the cerebellar microgyria seen along the mediolateral axis, the anterior–posterior foliation of the cerebellar vermis was significantly reduced in CHD7 knockout mice (Supplementary Fig. 4b). Consistently, the mediolateral length of the cerebellum was significantly increased whereas the anterior–posterior and dorsoventral lengths were significantly decreased following CHD7 depletion (Fig. 4e and Supplementary 4c). In histological analyses, Bergmann glial fibers and Purkinje cell bodies displayed a disorganized, irregular arrangement within their respective layers. Furthermore, while distinct molecular and granule cell layers were observed, the internal granule layer displayed altered thickness in CHD7-conditional knockout mouse cerebellum (Fig. 4f and Supplementary Fig. 4d, e).

To define the onset of cerebellar microgyria in conditional CHD7 knockout mice, we performed nano-CT scan of the cerebellum at distinct stages of development (Supplementary Fig. 3f). The pattern of cerebellar microgyria in adult conditional CHD7 knockout mice appeared by P7 (Supplementary Fig. 4g). Fine temporal resolution nano-CT-scanning in the postnatal period revealed that the onset of aberrant folding, marked by the emergence of a single sulcus at the mediolateral midpoint of the anterior cerebellar vermis, appeared at P3 in conditional CHD7 knockout mice (Fig. 4g). By P3.5, two symmetric microgyri had developed around the initial midline sulcus. Strikingly, by P4, four distinct microgyri had emerged in conditional CHD7 knockout mice (Fig. 4g). The location of each sulcus at P4 was exceptionally stereotyped among conditional CHD7 knockout mice (Fig. 4h). Remarkably, we identified indentations on the surface of the cerebellum in P3 control mice that spatially mimicked the location of each sulcus in conditional CHD7 knockout mice (Fig. 4g). However, in control mice the vermal indentations were transient, disappearing by the post proliferative stage of cerebellar development (Fig. 4g, h and Supplementary Fig. 4f, h). These data suggest that CHD7 regulates a window of cerebellar development that controls cerebellar foliation.

### CHD7 regulates axis of division of granule cell precursors in the cerebellum

**Fig. 5:**
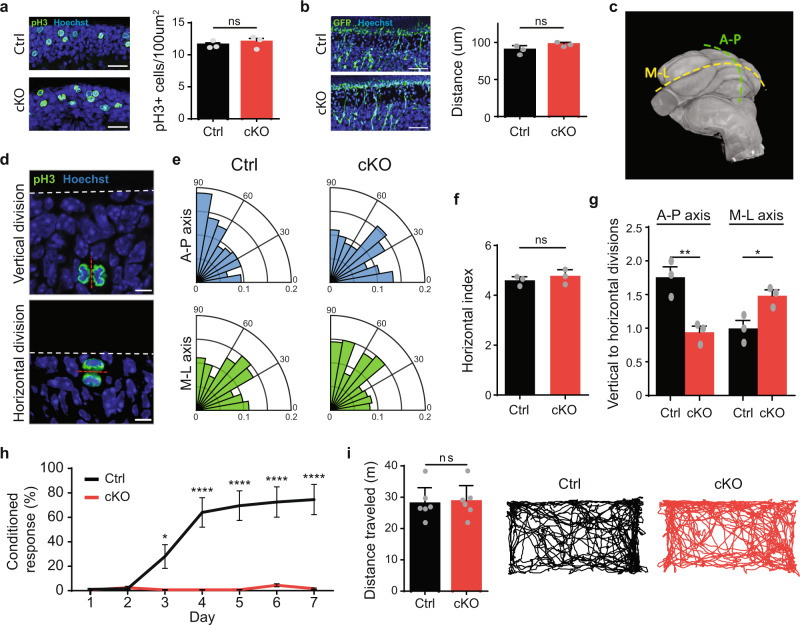
CHD7 regulates the preferred axis of division for GCPs. **a** Immunohistochemistry analysis of Ctrl and cKO mice (*n* = 3 for each condition) with antibodies recognizing phosphorylated histone H3 (pH3), a marker of dividing cells. Dividing cells were counted in the entirety of the region anterior to the primary fissure of the cerebellum (lobules I–V), referred to in shorthand as the anterior cerebellum. A representative image of the external granule layer is shown (left). Bar graphs (right) show mean pH3 positive cells per 100 µm^2^ ± s.e.m. ns = non-significant. Paired two-sided *t*-test. Scale bar: 25 µm. **b** P6 mouse pups were electroporated (*n* = 3 for each condition) with the GFP expression plasmid and killed 48 h later. Cerebella were removed, sectioned, and subjected to immunohistochemistry with antibodies recognizing GFP. Bar graphs show mean distance from EGL ± s.e.m. ns = non-significant. Paired two-sided *t*-test. Scale bar: 25 µm. **c** Image of P56 cerebellum obtained via nano CT scan (left) showing anterior–posterior axis (A–P) in green and medial–lateral axis (M–L) in yellow. **d** Immunohistochemistry analysis of axis of division of granule cell precursors, identified via pH3 staining in green. Dashed red line indicates the plane of division; dashed white line indicates the pial surface. Example of a granule cell precursor undergoing a vertical (top) and horizontal (bottom) division. Experiment was repeated three times for each condition. Scale bar: 5 µm. **e** Rose plot showing the distribution of axis of division angles (angle between pial surface and plane of division) for P3 Ctrl and cKO mice in A–P (top, blue) and M–L (bottom, green) axes. 180 cells (60 from each mouse, *n* = 3 for each condition) were analyzed for each rose plot. **f** Bar graph showing mean number of horizontal division per 100 µm^2^ ± s.e.m. (*n* = 3 for each condition). ns = non-significant. Paired two-sided *t*-test. **g** Bar graph showing mean ratio of vertical to horizontal division ± s.e.m. for Ctrl and cKO mice in both A–P and M–L axes (*n* = 3 for each condition). *p = 0.0068 (AP), *p* = 0.0256 (ML) Paired two-sided *t*-test. **h** Cerebellar-dependent eye-blink conditioning learning paradigm was performed on CHD7 Ctrl and cKO (*n* = 7 and *n* = 9, respectively) mice. Percent conditioned response (CR) is shown as mean ± SEM for each session day. ^∗∗∗∗^*p* < 10^− 4^, ^∗^*p* < 0.05 two-sided repeated-measures ANOVA, Sidak’s multiple comparison test. **i** Open-field assay was performed on CHD7 Ctrl and cKO mice (*n* = 6 for each) to assess general locomotor activity. Two-sided *t*-test; ns, not significant.

The consistent spatial and temporal features of microgyrification of the anterior cerebellum upon conditional CHD7 knockout led us to assess the cellular processes that might underlie the conditional CHD7 knockout phenotype. In immunohistochemical analyses of granule cell precursor proliferation using antibodies recognizing phosphorylated histone H3 (pH3), conditional CHD7 knockout had little or no effect on the rate of granule cell proliferation in the anterior or posterior cerebellum in P4 mice (Fig. 5a and Supplementary Fig. 5a). In other analyses, in vivo electroporation of GFP-expressing plasmid into granule cell precursors of P4 mouse cerebellum revealed little or no effect on neuron migration from the EGL to the IGL upon conditional CHD7 knockout (Fig. 5b). In addition, conditional CHD7 knockout had little or no effect on granule neuron polarity during migration or dendrite development in vivo (Supplementary Fig. 5b, c). Altogether, these results suggest microgyrification upon CHD7 depletion occurs at a time when postnatal granule cell proliferation rate is normal, and that migration and neurite development is not an underlying driver of these effects.

The normal anterior–posterior foliation of the cerebellum is thought to arise from preferential division orientation of granule cell precursors along the anterior–posterior axis of the cerebellum^31,32^ (Fig. 5c). Therefore, we asked whether orientation of granule cell precursor division is disrupted in conditional CHD7 knockout mice. Immunohistochemical analyses of mediolateral and anterior–posterior sections of the cerebellum revealed granule cell precursors in anaphase with orientation of cell division in a horizontal (parallel to the EGL surface), vertical (perpendicular to the EGL surface) or oblique plane (Fig. 5d). In these analyses, we observed a higher ratio of vertical to horizontal divisions in the anterior–posterior plane compared to the mediolateral plane in control mice (Fig. 5e, g), consistent with the interpretation that anterior–posterior folding of the cerebellum in control mice arises from preferential division of granule cell precursors in the anterior–posterior plane. Remarkably, the ratio of vertical to horizontal divisions was reduced in the anterior–posterior plane and concomitantly increased in the mediolateral plane in conditional CHD7 knockout mice (Fig. 5e, g), suggesting that CHD7 depletion triggers a shift in the axis of granule cell precursor division from a normally anterior–posterior orientation to a preferentially mediolateral orientation in conditional CHD7 knockout mice. In other analyses, conditional CHD7 knockout had little or no effect on the number of horizontal divisions in both anterior–posterior and mediolateral sections of the cerebellar cortex, consistent with lack of effect of CHD7 depletion on the rate of granule cell precursor proliferation at the timepoint analyzed (Fig. 5f). Collectively, our results suggest that CHD7 plays a critical role in regulation of granule cell precursor division orientation, providing a potential cellular basis for cerebellar microgyria in conditional CHD7 knockout mice.

Recent studies have postulated a model whereby cerebellar folding arises from differential expansion between an outer layer of proliferating granule cell precursors expanding along the anterior–posterior axis at a faster rate than the slowly enlarging inner core of the cerebellum^33^. Our observation of a mediolateral shift in the orientation of granule cell precursor division in conditional CHD7 knockout mice led us to ask whether differential expansion occurred in the mediolateral axis. Remarkably, expansion of the mediolateral axis of the cerebellum was increased in conditional CHD7 knockout mice, which coincided with the appearance of cerebellar microgyria (Supplementary Fig. 5d). Thus, CHD7 depletion may result in the formation of cerebellar microgyria due to the deregulation of cellular processes that normally contribute to cerebellar foliation during perinatal brain development.

As structural abnormalities of the cerebellum might be associated with deficits in cerebellar processing in the adult brain^34–36^, we asked whether conditional knockout of CHD7 might impact cerebellar function in adult mice. We subjected conditional CHD7 knockout mice and control littermates to a delayed eye-blink conditioning response paradigm of associative motor learning. Conditional CHD7 knockout mice displayed impaired cerebellar-dependent eye-blink conditioning (Fig. 5h). In other behavioral analyses, CHD7 depletion had little or no effect on general motor coordination and ambulatory activity, respectively assessed by DigiGait assay and open-field test (Fig. 5i and Supplementary Fig. 5e). These results suggest that CHD7 function in granule cell precursors of the developing anterior cerebellum robustly influences motor learning in adult mice. Although CHARGE patients are not known to display classic features of cerebellar dysfunction, our findings suggest that additional investigations into the role of CHD7 in cerebellar function may warranted.

### CHD7-regulated cerebellar tissue morphogenesis genes are evolutionarily conserved

**Fig. 6:**
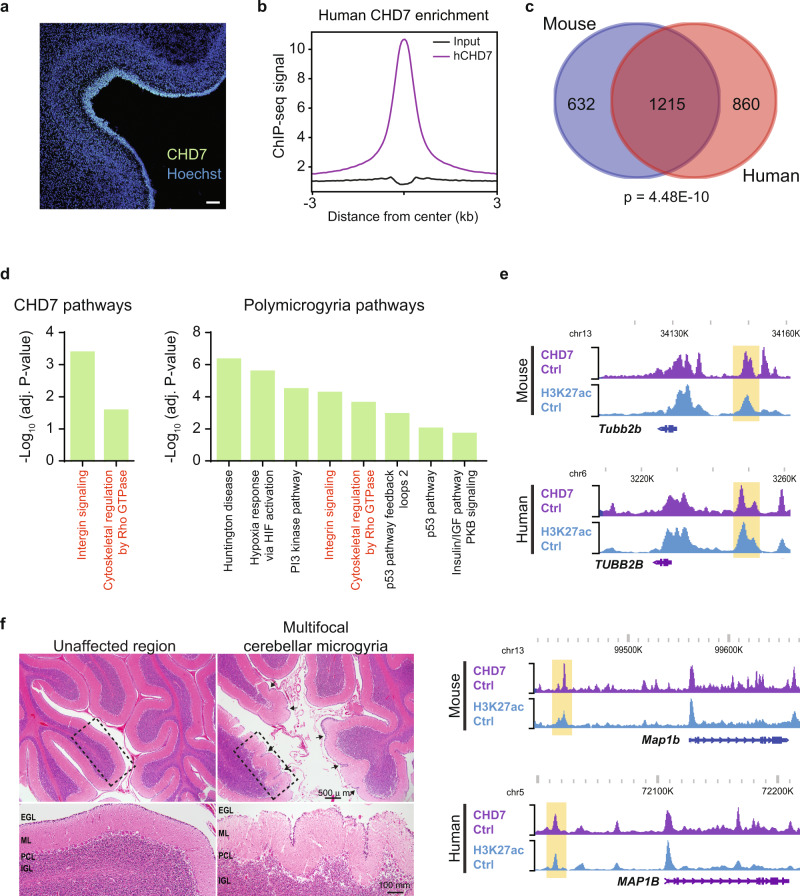
CHD7-bound genes implicated in brain folding disorders are significantly conserved between perinatal mouse and fetal human cerebellum. **a** Immunohistochemistry analysis of 22 gestational week human cerebellum with antibodies recognizing hCHD7. Experiment was performed once on multiple sections. Scale bar: 100 µm. **b** Aggregate plot of ChIP-seq signal for hCHD7 (purple) and input (black) centered on hCHD7 genomic binding sites (*n* = 17,100). 22 gestational week anterior human cerebellum (*n* = 1 for CHD7 Ctrl, *n* = 1 for input). **c** Venn-diagram showing overlap of CHD7 mouse and human target genes with hypergeometric analysis. *p*-value =  4.48E-10, hypergeometric. See Methods section for more details on analysis. **d** Panther pathways analysis of mouse CHD7 direct target genes (left) and PMG-associated genes (right). Shared pathways are shown in red. **e** WashU Epigenome Browser view of two CHD7 direct target genes (TUBB2B, top; MAP1B, bottom) found in mouse and human cerebellum. Each site shows ChIP-seq coverage of CHD7 and H3K27ac. **f** H&E staining of 5-month-old clinically diagnosed CHARGE syndrome patient. Arrowheads point to sites or excessive ectopic cerebellar foliation. Zoom in of dashed rectangle shown below. EGL external granule layer; ML molecular layer; PCL Purkinje cell layer; IGL internal granule layer. Experiment was performed once on multiple sections for each region. Please refer to Methods for additional details regarding patient cerebellar sample.

We next determined if CHD7 regulation of chromatin and folding in the cerebellum is conserved in the human cerebellum. We assessed the potential role of CHD7 in human cerebellar development at a stage that corresponds to early stages of cerebellar development in mice. Immunohistochemical analysis of 22-week gestational age human cerebellum revealed the EGL delineated by Pax6-positive granule cell precursors superficial to cell bodies of Calbindin-positive Purkinje cells and S100β-positive Bergmann glia (Supplementary Fig. 6a). The expression of CHD7 co-localized with Pax6-immunoreactivity in the EGL in the human cerebellum (Fig. 6a), suggesting that as in mouse cerebellum, CHD7 functions in granule cell precursors in the human cerebellum.

To identify putative gene targets of CHD7 in the developing human brain, we performed ChIP-seq of CHD7 in the cerebellum of 22-week gestational age fetal tissue (Fig. 6b and Supplementary Fig. 6b). These analyses revealed significant overlap between human and mouse CHD7 gene targets (Fig. 6c). Approximately 66% of genes associated with CHD7-binding sites in mouse cerebellum were also associated with CHD7 in the human fetal cerebellum (Fig. 6c). H3K27ac ChIP-seq analyses suggested that human and mouse CHD7 similarly occupied regulatory elements containing this activation-associated mark (Supplementary Fig. 5b). Gene ontological analysis of genes bound by mouse and human CHD7 revealed enrichment for several molecular pathways involved in intercellular communication and hormone signaling (Fig. 6d).

The role for CHD7 in cerebellar tissue morphogenesis in mice led us to evaluate whether conserved CHD7-direct target genes were enriched in molecular pathways implicated in aberrant brain folding. Remarkably, the two significantly enriched CHD7-regulated pathways were identified as polymicrogyria-enriched molecular pathways, which included integrin signaling and cytoskeletal regulation by Rho GTPase (Fig. 6d and Supplementary Data 1). For example, both mouse and human CHD7 occupied an active enhancer upstream of the *Tubb2b* and *Map1b* genes (Fig. 6e), and each of these enhancers was found to be within TADs that contained these genes in both mouse and human (Supplementary Fig. 6c). Notably, mutation of the *Tubb2b* and *Map1b* genes have been implicated in polymicrogyria in the cerebral cortex^37,38^. Consistent with these observations, assessment of cerebellar morphology in a patient with clinically diagnosed CHARGE syndrome, the only one with available cerebellar tissue for examination, revealed excessive cerebellar cortical folding suggestive of cerebellar microgyria (Fig. 6f). Histological analysis identified intact cerebellar cortical layering in regions of pronounced folding (Fig. 6f), similar to the cerebellar microgyria in conditional CHD7 knockout mice. While these data support conserved microgyria due to CHD7 disruption across mouse and humans, additional investigation of human brain samples from patients with confirmed CHD7 mutations in the future will be required to confirm its significance in CHARGE syndrome. These data suggest a conserved genome regulatory function for CHD7 that may contribute to the proper folding of mouse and human cerebellum, with potential implications for our understanding of cerebellar deficits in CHARGE syndrome.

## Discussion

In this study, we have defined the role of CHD7 in the regulation of epigenomic modifications associated with enhancer activation in granule cell precursors and consequent morphogenesis of the cerebellar cortex. Using ATAC-seq and ChIP-seq analyses of the anterior cerebellum in conditional CHD7 knockout mice, we have found that CHD7 robustly promotes accessibility, active histone modifications, and recruitment of RNAPII of enhancers in granule cell precursors. Remarkably, genome architecture analyses of the anterior cerebellum reveal that CHD7-dependent enhancer activation correlates with local alterations of chromatin. Genome and gene ontology studies show that CHD7-regulated enhancers and genes in granule cell precursors prominently include cerebellar morphogenesis genes. Accordingly, we have discovered that conditional CHD7 knockout triggers a striking cerebellar polymicrogyria phenotype, which we have also found in a case of CHARGE syndrome. These findings elucidate CHD7 functions in gene enhancer regulation and tissue morphogenesis in the cerebellum, with potential implications for our understanding of CHARGE syndrome.

Prior to our study, a role for CHD7 in the regulation of chromatin accessibility had been proposed but how the activity of the protein impacts other epigenetic modifications associated with the poising and activation of enhancers remained largely unexplored. We have found that CHD7-bound accessible enhancers bear the developmentally distinct epigenomic states of active and poised enhancers. Studies of mouse embryonic stem cells suggest that poised enhancers, which are accessible yet lack definitive marks of active regulatory regions^39,40^, may be in a state of readiness for activation in a time- and lineage-specific manner^26,27^. We have found that CHD7-bound poised enhancers undergo dynamic changes in epigenomic state during cerebellar development, whereby CHD7-bound enhancers implicated in neuronal differentiation acquire histone modifications associated with enhancer activation during later phases of cerebellar development. Correspondingly, deregulation of genes implicated in neuronal differentiation occurs at a more advanced stage of cerebellar development in conditional CHD7 knockout mice^16^. In parallel, we demonstrate that during an earlier stage of cerebellar development CHD7 regulates the accessibility, histone acetylation and RNAPII binding at enhancers implicated in cerebellar morphogenesis. Collectively, these data suggest that CHD7 governs multiple phases of cerebellar development through regulation of enhancers characterized by temporally distinct epigenomic states. In addition, while our findings are consistent with previous work predicting the targeting of CHD-family chromodomains to H3K4me^41^ and later demonstrating the preferential association of CHD7 with H3K4me1-positive enhancers^42^, whether CHD7 plays a broad role in regulating the presence of the poised mark H3K4me1 at enhancers had remained unclear prior to our study. Our findings of limited changes in H3K4me1 at CHD7-bound enhancers, in contrast to more pronounced changes in active epigenomic marks H3K27ac and RNAPII, suggests that CHD7 may play a limited role in the poising of enhancers while having a greater influence on enhancer activation. Notably, the occupancy of CHD7 at poised enhancers that lack histone acetylation suggests that its activating role might be dynamically regulated. Such regulation as well as dynamic changes in CHD7 binding offer an epigenetic basis for the diverse array of spatiotemporally specified functions regulated by ATP-dependent chromatin remodelers.

Enhancers coordinate specific transcriptional programs through multiple mechanisms^24,43,44^. The architecture of chromatin is a fundamental component of a multilayered epigenetic regulatory program that spatially facilitates or restricts the regulatory interactions of enhancers on gene expression^45–47^. Hi-C analyses upon CHD7 depletion reveal that TADs and compartment structures remain remarkably unchanged, with only modest changes of distal regulatory interactions are observed. Nonetheless, CHD7 regulation of enhancer accessibility results in corresponding changes in gene expression at promoters spatially linked by TAD structures and E–P loops. While correlative, these data support a model in which CHD7 alters local chromatin to stimulate enhancer activity. By contrast, the CHD-family member CHD4 concordantly regulates enhancer accessibility and genome architecture^17^, suggesting that CHD7 activity at enhancers may regulate transcriptional programs in a manner distinct from related CHD proteins. Consistent with a more specific role of CHD7 in enhancer regulation, changes in gene expression and histone modifications may occur in the absence of alterations in largescale chromatin organization^43,48–50^. Thus, our analyses associate downregulation of gene expression with decreased accessibility upon CHD7 depletion, which, in addition to its corroboration by prior studies, provides a new level of resolution and mechanistic understanding for how CHD7 affects epigenomic states at enhancers and impacts gene expression.

By comprehensively assessing CHD7 binding, accessibility, chromatin organization and transcription, we uncover a role for CHD7 in the regulation of genes implicated in cerebellar morphogenesis during early perinatal cerebellar development. This finding is accompanied by the striking formation of cerebellar microgyria in conditional CHD7 knockout mice. The highly stereotyped and fully penetrant folding defect in conditional CHD7 knockout mice offers a system to characterize the molecular and cellular processes underlying brain folding. We provide in vivo experimental evidence supporting a role for orientation of granule cell precursor division in uniaxial folding of the cerebellum. Consistent with our findings, granule cell precursor clonal analysis and mechanical modeling have raised the hypothesis that folding of the brain may arise from compressive forces due to differential expansion between the outermost layer of the brain undergoing a high rate of tangential expansion relative to a more slowly growing inner zone^32,33,51–53^. In addition to its perinatal function, CHD7 also plays a temporally distinct role during embryogenesis^54^. During embryogenesis, CHD7 regulates the expansion of granule cell precursors by maintaining high levels of FGF8 in the mid-hindbrain organizer^13,55^. Diminished FGF signaling contributes specifically to hypoplasia of the cerebellar vermis^56^. Prior studies of conditional CHD7 knockout mice have identified cerebellar hypoplasia in the setting of altered granule cell precursor proliferation in the anterior lobules at or before P0 but, in agreement with our findings, do not detect such proliferation deficits at later postnatal developmental timepoints in the anterior cerebellum^15,16^. Future studies examining the developmental cues regulating embryonic and postnatal proliferation of granule cells could shed more light on the temporally restricted control of granule cell proliferation by CHD7.

Thus, CHD7 depletion appears to affect cerebellar development in two distinct phases. CHD7 loss results in the embryonic reduction of cerebellar expansion leading to cerebellar hypoplasia. Perinatally, CHD7 loss leads to reorientation of granule cell precursor division plane in the EGL. Whether the reorientation of granule cell precursor division is completely independent of perturbed embryonic granule cell precursor proliferation or stems in part from this earlier effect remains to be determined. Nonetheless, these consecutive insults may combinatorically exacerbate differential expansion to create mediolateral folds. Specifically, increased mediolateral expansion of the EGL may represent an outer zone expanding at a significantly higher rate relative to a hypoplastic inner core produced during embryonic development.

Although the preferred orientation of division offers a potential cellular basis for the uniaxial lobulation of the cerebellum, the spatial stereotypy of fissures is thought to arise from anchoring centers, identified by indentations on the surface of the developing cerebellum^57^. Changes in behavior of granule cell precursors may drive the formation of anchoring centers at the base of each sulcus along the anterior–posterior axis^33,57^. In our study, CT-scanning coupled with immunohistochemical analysis of developing cerebellum have identified transient vermal indentations along the mediolateral axis. Whether these indentations represent mediolateral anchoring centers will require additional studies to thoroughly characterize the cytoarchitectural features of these transient sites. Increased mediolateral expansion upon CHD7 depletion superimposed on mediolateral anchoring centers may mediate formation of stereotypic mediolateral folding in conditional CHD7 knockout mice. Altogether, these observations suggest that folding anomalies may arise from deregulated cellular processes that rearrange pre-existing architectural features in the brain.

Understanding cerebellar folding is critical due to the association of cerebellar structural abnormalities with human neurodevelopmental disorders of cognition. A preponderance of empirical evidence suggests cerebellar participation in multiple domains of human cognition and perception, including implicit learning and predictive processing^58^. Notably, differences in cerebellar structure may be associated with autism^34–36^. The mouse cerebellum may serve as a useful model to understand structural anomalies in human cerebellum given the relatively conserved folding pattern between these species^33^. We reveal significant conservation at the genomic level by identifying significant conservation between mouse and human CHD7-regulated genes in the developing cerebellum. Conserved CHD7-regulated genes control pathways, including integrin signaling and cytoskeletal regulation by RhoGTPase, implicated in polymicrogyria, a disorder of excessive folding. Whereas most genes implicated in human folding disorders have focused on effectors of cytoskeleton and cell-adhesion, these data implicate chromatin remodelers in the regulation of proper folding. Interestingly, the conserved gene pathways identified in our study have also been implicated in the regulation of axis of division. As the molecular underpinnings of these complex cellular and anatomical processes remains largely uncharacterized, future studies should allow for a more targeted assessment of relevant CHD7 downstream effectors. In sum, our study uncovers potential molecular and cellular mechanisms by which chromatin remodelers contribute to brain morphogenesis during development and disease.

## Methods

### Mice

Mice were maintained in a pathogen-free environment. Mice were housed in a 12 h on, 12 h off light cycle with room temperature between 20 and 26 °C and humidity between 30–70%. All procedures involving animals were performed according to protocols approved by the Animal Studies Committee of Washington University School of Medicine and in accordance with both the National Institute of Health Standings Committee on Animals as well as the National Institutes of Health guidelines. CHD7 fl/fl and Atoh1-Cre have been described^59^ (CHD7 fl/fl: Hai Kun Liu; ATOH1-Cre: Matei et al.^60^). For all experiments, control mice are double floxed littermate mice without the Atoh1-Cre transgene. Please refer to Supplementary Table 2 for a list of genotyping primers.

### Antibodies

Antibodies to CHD7 (Immunofluorescence 1:2000, Immunoblot 1:1000, ChIP 1:100, Cell Signaling Technology, #6505 S), GAPDH (Immunoblot 1:500, Santa Cruz, #sc-32233), histone H3K27ac (ChIP 1:1000, Active Motif, #39133), H3K4me1 (ChIP 1:250, Active Motif, #39297), H3K4me3 (ChIP 1:125, Cell Signaling Technology, #9751 S), RNAP2 (ChIP 1:10, Santa Cruz, #sc-47701), CTCF (ChIP 1:200, Millipore, #07-729), RAD21 (ChIP 1:200, Abcam, #ab992), Calbindin (Immunofluorescence 1:5000, Swant, D-28K), GFP (Immunofluorescence 1:250, Abcam, #ab13970), GFAP (Immunofluorescence, Abcam, #ab7260), phospho-Histone H3 (Immunofluorescence 1:1000, Millipore, #06-570), S100β (Immunofluorescence 1:250, Abcam, #ab41548) were purchased. Alexa fluor conjugated secondary antibodies were utilized for immunofluorescence (1:500, Thermo Fisher Scientific, #A-11008, A-11010, A-11011, A-11012). Antibodies to CHD7 (ChIP, IP, IB) have been described^15,16^.

### Immunohistochemistry

The cerebellum from mice and human were fixed with 4% PFA and 4% sucrose and subjected to cryo-sectioning on the Leica CM3050S Cryostat. Sections were blocked with blocking buffer (10% goat serum, 3% BSA, and 0.4% Triton X in PBS). Subsequently, sections were incubated overnight with relevant primary antibodies followed by a 2-h incubation with Alexa Fluor conjugated secondary antibodies. The DNA dye Bisbenzimide (Hoechst 33258) was used to label cell nuclei. Confocal images were acquired with a Zeiss LSM 880 II Airyscan FAST Confocal Microscope or an Olympus FV1200 Confocal Microscope.

### GCP axis of division

Orientation of GCP division was compared in the cerebellum of P3 littermate conditional CHD7 knockout and control mice, as described^31^ with minor modifications. In both coronal (mediolateral) and sagittal (antero-posterior) sections of the cerebellum, cells in anaphase were classified as a horizontal division (parallel to the EGL surface), vertical division (perpendicular to the EGL surface) or oblique division (see Fig. 5c, d for depiction of sectioning planes and division orientations). Cells in anaphase were detected by antibodies recognizing phosphorylated histone H3 (Millipore, #06-750), a marker of dividing cells. In coronal sections, vertical divisions are thought to lead to cellular expansion along the mediolateral axis of the cerebellum, whereas in sagittal sections, vertical divisions are thought to lead to expansion along the anterior–posterior axis. By contrast, a horizontal division contributes to thickening of the EGL in both sections of the cerebellum, as it divides in a direction perpendicular to the pia mater. Thus, horizontal divisions were used as a normalizing factor when assessing differences in vertical divisions between coronal and sagittal sections.

### Delay eye-blink conditioning

Delay eye-blink conditioning assay was adapted from the procedure used by Heiney et al.^61^. Littermate conditional CHD7 knockout (*n* = 4 males and 5 females) or control (*n* = 3 males and 4 females) mice at 8 weeks of age were used. Prior to head plate implantation surgery, mice were first anesthetized with ketamine/xylazine (100 mg/kg; 10 mg/kg). Skin overlying the skull was cleaned with betadine and alcohol scrub. Skull was exposed by a full-thickness midline incision followed by clearance of the underlying fascia with cotton swabs. Two small screws were symmetrically placed on both sides of the midline near bregma. A thin-metal head plate was then placed over bregma with the screws fitting along the inner border of the central hole of the head plate. The head plate was stabilized using Metabond cement (Parkell) over the Bregma skull landmark^62^. After 5 days of post-surgical recovery, head-fixed mice underwent two consecutive days of 1 h habituation sessions on a cylindrical treadmill. After training, mice underwent experimental testing in the head-fixed eye-blink conditioning apparatus. In the paradigm, mice gradually associate a conditioned stimulus (CS; blue LED) with an eye-blink-eliciting unconditioned stimulus (US, 20 psi periocular air puff through a 25-gauge needle; CS-US inter-stimulus interval, 150 msec). One-hundred trials of CS-US pairings were performed each day over 6 consecutive days. The learned eyelid conditioned response was recorded using a high-speed monochrome camera (Allied Vision). Fraction of eyelid closure, ranging from 0 (fully open) to 1 (fully closed), was calculated on each frame by the pixel area method^61^. During the inter-stimulus period, eyelid closure >0.1 was designated as a conditioned eyelid response (CR). Our measure for motor learning was the percentage of CR-positive trials on each session day (Percent CR).

### DigiGait analysis

The DigiGait imaging platform (Mouse Specifics Inc, Quincy, MA, USA) was employed to assess gait dynamics in littermate 5-week-old conditional knockout (*n* = 7 males and 8 females) and control mice (*n* = 4 males and 7 females)^63–65^. During mouse ambulation on a transparent treadmill (20 cm/s, 0° incline), digital paw prints were captured by high-speed camera. Each mouse was tested individually on the treadmill, which was enclosed by a polycarbonate compartment of 5 cm width and 25 cm length. Changes in the area of contact for each paw as it was being placed on the belt and it was being lifted from it during a step were calculated. Gait signal for each of the four limbs was then utilized to quantify and analyze gait-related variables by software specialized for the DigiGait imaging system. Approximately 5 s of video were collected from each mouse to provide an adequate number of sequential strides for quantification of stride-related variables.

### In vivo electroporation

In vivo electroporation of postnatal mouse pups was performed^3,66–69^. P6 littermate CHD7-conditional knockout and control littermate mouse pups were lightly anesthetized (isoflurane 5% in O_2_ for induction, 1–2% for maintenance) prior to surgically revealing the injection site. A small incision was made in the dorsal scalp to expose occipital bone overlying the cerebellum, which was targeted for midline injection. All mice were injected with pCAG-GFP, and subjected to four electric pulses of 135 mV with 950 ms intervals. Incisions were closed with suture. Electroporated pups were returned to moms and examined in a blinded manner by immunofluorescence confocal microscopy 2 days later for migrations analysis and 8 days later for dendrite number analysis.

### X-ray microscopy

The cerebellum from mice were stained with Lugol’s Iodine. Cerebella were imaged in Zeiss Versa 520 X-ray microscope using 0.4x flat panel detectors to analyze tissues. Analysis of three-dimensional (3D) and two-dimensional (2D) images were performed with Dragonfly (v4.0 Object Research Systems). For comparative measurement of cerebellar length, nano-CT scan was performed for three biological replicates of sex-matched control littermate Ctrl (*n* = 2 males and 1 female) and cKO (*n* = 2 males and 1 female) p56 mouse cerebella. Subsequently, Dragonfly imaging software was utilized to identify the longest mediolateral axis length of the 3D composite image of the entire cerebellum (lobules I–X). Measurement of the longest mediolateral axis length for the anterior cerebellum was performed similar to that for the entire cerebellum, but instead, lobule V was measured. The longest mediolateral axis for the entire cerebellum and anterior cerebellum were verified by measuring the mediolateral axis of 2D coronal images.

### RNA-sequencing

For RNA-seq, total RNA was extracted from the anterior cerebellar vermis of P4 littermate mice using RNeasy Plus Micro Kit (Qiagen) with gDNA eliminator column. Two-hundred nanograms of total RNA for each experimental sample underwent depletion of rRNA utilizing the NEBNext rRNA Depletion Kit (NEB). Quality control performed using Agilent Technologies Bioanalyzer. Libraries were then prepared from intact RNA utilizing the NEBNext Ultra II Directional RNA Library Prep Kit for Illumina (NEB). Sequencing of libraries performed on Illumina HiSeq 2500 (Genome Technology Access Center). Five biological replicates were sequenced in all experiments.

### Chromatin immunoprecipitation

ChIP-seq was performed as previously described with minor modifications (Lingchun Kong, A Primary Role of TET Proteins in Establishment and Maintenance of De Novo Bivalency at CpG Islands). P4 anterior cerebellum were dissected and pinched to 1–3 mm^3^ pieces with tweezers, then fixed in 1% formaldehyde solution in 1x PBS for 15 min at room temperature. Formaldehyde was quenched with 125 mM of glycine for 5 min at room temperature. Tissues were washed three times with cold 1x PBS/0.1% NP-40/PMSF, then homogenized with loose Dounce. Cells were filtrated through a 40 µm cell strainer. As described in Majidi and Reddy et al.^70^, P22 cerebellum was dissected, homogenized 20 times with loose dounce followed by 12 times with tight dounce in 1% formaldehyde solution in 1x PBS, and incubated for 15 min. Formaldehyde was quenched with 125 mM of glycine for 5 min at room temperature. Tissues were washed three times with cold 1x PBS/PMSF. For nuclear purification, tissue was resuspended in Lysis Buffer 1 (50 mM HEPES-NaOH pH 7.5, 140 mM NaCl, 1 mM EDTA, 1 mM EGTA, 0.25% Triton X-100, 0.5% NP-40, 10% Glycerol), homogenized four times with tight dounce, and incubated for 10 min at 4 °C. Tissue was then resuspended in Lysis Buffer 2 (10 mM Tris-Cl pH 8.0, 200 mM NaCl) and incubated for 10 min at 4 °C. Finally, the tissue was resuspended in cold Lysis Buffer 3 (10 mM Tris-Cl pH 8.0, 100 mM NaCl, 1 mM EDTA, 0.5 mM EGTA, 0.1% Na-Deoxycholate, 0.5% N-Lauroylsarcosine). Chromatin was fragmented in Lysis Buffer 3 with 0.1% SDS using the Covaris E220 sonicator (5% Duty Factor, 140 Peak Incidence Power, 200 cycles per burst, milliTUBE 1 mL AFA Fiber). One million cells were used for each immunoprecipitation reaction. For H3K4me1, H3K4me3, and CTCF ChIPs, Sepharose protein G and protein A (GE Life Sciences) were used; for H3K27ac and RAD21 ChIPs, Dynabeads protein G and protein A (ThermoFisher Scientific) were used; for RNA PolII ChIP, Dynabeads protein G (ThermoFisher Scientific) were used. ChIP-seq libraries were prepared using the Swift NGS 2S Plus Library Prep Kit per kit instructions, then sequenced on an Illumina NextSeq 500 to obtain 75 bp single-end reads (Center for Genome Sciences at Washington University). Three biological replicates for ChIP-seq experiments.

### ATAC-seq

P4 anterior cerebellums were dissected and washed with cold 1x PBS, then homogenized in cold ATAC lysis buffer (10 mM HEPES, pH 7.4, 10 mM NaCl, 1.5 mM MgCl2, 0.1% IGEPAL CA-630) with tight Dounce. Nuclei were filtered with a 40 µm cell strainer. In all, 50,000–100,000 nuclei were washed with 500 µL of cold ATAC lysis buffer then pelleted by gentle centrifugation. Nuclear pellets were resuspended in 50 µL of transposition reaction mix (25 μL of 2x TD Buffer (Illumina, FC-121-1030), 5 µL of Tn5 Transposase (Illumina, FC-121-1030), 5 µL of 0.1% Digitonin, 5 µL of 0.2% Tween-20 and 10 µL of H_2_O). The transposition reaction mix is incubated at 37 °C for 30 min in a thermomixer set at 800–1000 rpm. DNA was purified with Qiagen MinElute PCR kit and amplified using 10 cycles of PCR reaction (25 µL of DNA, 5 µL of 10 µM Custom Adapter Mix i5 and i7, 10 µL of 5x Reaction Buffer, 1.5 µL of 10 mM dNTPs, 0.5 µL of Q5 Polymerase (NEB), 8 µL of H2O). The amplified DNA was purified with Beckman Coulter SPRI beads and sequenced on the Illumina NextSeq 500 platform to obtain 40 bp paired-end reads (Center for Genome Sciences at Washington University). Three biological replicates for ATAC-seq experiments.

### Hi-C

Hi-C was performed as previously described with minor modifications^17^ (Lieberman-Aiden et al.^71^).

#### Crosslinking

P4 anterior cerebellums were dissected and pinched to 1–3 mm^3^ pieces with tweezers, then fixed in 1% formaldehyde solution in 1x PBS for 15 min at room temperature. Formaldehyde was quenched with 125 mM of glycine for 5 min at room temperature. Tissues were washed three times with cold 1x PBS/0.1% NP-40/PMSF, then homogenized with loose Dounce. Cells were filtrated through a 40 µm cell strainer. In all, 500,000 cells were used for each Hi-C reaction.

#### Chromatin clean

Cell pellets were resuspended in cold Hi-C lysis buffer (10 mM HEPES 7.4, 10 mM NaCl, 1.5 mM MgCl2, 0.2% NP-40), then incubated on ice for 15 min. Cells were centrifuged, then resuspended in 0.5% SDS and incubated at 65 °C for 6–7 min to inactivate proteins.

#### Chromatin digestion and in situ ligation

SDS was diluted and quenched with Triton X-100 at 37˚C for 15 min. Nuclei were then treated with 50 U of MboI (NEB) in 1x NEBuffer 2.1 and incubated at 37 °C for 3 h, followed by incubation at 65 °C for 20 min to inactivate the enzyme. DNA blunting was performed by incubating nuclei with 5 nmol of Biotin-14-dATP and other dNTPs with 20 U of Klenow (NEB) at 37 °C for 1 h. Proximity ligation was performed by incubating nuclei with 200 U of T4 DNA Ligase (NEB) in a ligation buffer (1x T4 DNA ligase buffer, 1% Triton X-100) for 3 h at room temperature. Nuclei were centrifuged, then resuspended in de-crosslink buffer (50 mM Tris, pH 8.0, 10 mM EDTA, 1% SDS) and incubated at 65 °C for 4 h-overnight.

#### DNA purification and removal of biotin from free ends

Twenty micrograms of Rnase A and 40 µg of Proteinase K were used to digest RNA and protein. DNA was purified by Phenol–Chloroform purification. Biotin was removed from free ends in Biotin-removal mix (10 µL of 10× NEBuffer 2.1, 1 µL of 10 mM dGTP, 39 µL of H2O, 49 µL of DNA and 1 µL of T4 DNA polymerase (3 U/ul, NEB)) for 2 h at room temperature. The reaction was stopped by 10 mM EDTA DNA.

#### DNA shearing, biotin-capture, and preparation for Illumina sequencing

DNA was sonicated to 300 bp using a Covaris E220 instrument, then Biotin-labeled DNA was captured using Dynabeads MyONE Streptavidin T1 (Invitrogen). Beads were resuspended in 60 µL of pre-mixed Repair I Master Mix (SWIFT, ACCEL-NGS® 2S PLUS DNA LIBRARY KITS) (53 µL of low-EDTA TE, 6 µL of Buffer W1 and 1 µL of Enzyme W2). The following library was prepared using the Swift NGS 2S Plus Library Prep Kit per kit instructions with minor modifications. The beads were washed with 1x TBW buffer (10 mM Tris pH 8.0, 1 mM EDTA, 1 M NaCl, 0.05% Tween-20). Prior to amplification, DNA was eluted from beads by incubation in Low-EDTA TE at 98 °C for 10 min. DNA was then amplified using 9–10 cycles of PCR according to kit instructions. Following amplification, DNA was sequenced on the NextSeq 500 platform to obtain 75 bp paired-end reads (Center for Genome Sciences at Washington University). Three biological replicates were used for Hi-C experiments.

### Patient cerebellar sample

Diagnosis of this patient at 5 months of age was based on hallmarks of CHARGE, including choanal atresia, multiple heart defects, genitourinary abnormalities, and triangular conchae of the ear. Each of these findings on its own is relatively rare, and when seen together become highly specific for CHARGE. In addition, the patient received the available genetic workup procedures at the time, for example, to rigorously rule out DiGeorge Syndrome, a more prevalent disorder that shares some clinical features with CHARGE. Fluorescent in situ hybridization studies were negative for the 22q11.2 locus for DiGeorge Syndrome, as were chromosomal analyses for genetic defects. We attempted DNA extraction from formalin fixed paraffin embedded tissue from this patient in order to perform sequence analysis of the CHD7 gene; however, we were unable to do so as the tissue is 24.5 years old, and the DNA is likely degraded. On gross pathologic examination, multiple practicing neuropathologists observed frank polymicrogyria of the left middle lobe, including both the biventral and inferior semilunar lobules. Given the clear diagnosis of CHARGE and that an estimated 67–90% of CHARGE patients have mutations in CHD7^14,72,73^, we report this case as a relevant initial finding, and propose that analyses of postmortem brain tissue from CHD7 mutant CHARGE patients by investigators with access to the tissue is warranted.

### Statistical analysis

Statistical analysis for each experiment is detailed in the figure legends. Statistical significance for enrichment was evaluated using a two-tailed Chi-squared test/hypergeometric test. Box-whisker plots display median value with whiskers representing the 5th and 95th percentile. Significance testing for box-whisker plots were performed using two-tailed unpaired *t*-test or ANOVA with Tukey multiple comparison test, when appropriate. CHD7-regulated genes were defined as the single nearest gene within the same TAD and within 100 kb of a CHD7 ChIP-seq peak. Similarly, enhancers were mapped to their single nearest gene located within the same TAD and within 100 kb upstream or downstream of the enhancer. For behavioral experiments, independent *t*-test and repeated measures ANOVA followed by Sidak’s multiple comparison correction were used when appropriate. Threshold for calling statistical significance for all analyses mentioned above was *p* < 0.05. Statistical analyses were performed using GraphPad PRISM 6.0 and R (v3.5.1). Heatmaps were generated using DeepTools (v3.3.0). Please refer to Supplementary Table 1 for the number of reads per sample for all next-generation sequencing experiments, including ChIP-seq, RNA-seq, ATAC-seq, and Hi-C datasets.

### ChIP-seq alignment and peak calling

Single-end reads of 50 or 75 base pairs were obtained for all datasets. Samples were sequenced to a minimum depth of 18.5 million reads and aligned to the mm10 genome using Bowtie2 (v2.3.4.2) with default parameters for Galaxy platform. Reads were then filtered for a map quality score greater than 10 (mapQuality >10). Post-alignment processing and analysis was performed with Samtools (v1.8), Deeptools (v3.3.0), Bedtools (v2.29.0). Peaks were called using MACS2 (v2.1.1.20160309) on individual replicates. All peaks identified in at least two control or conditional knockout samples were used in downstream analyses. Regarding residual CHD7 ChIP signal in CHD7 conditional knockout mice, we offer the following rationale. Within the cerebellum, expression of CHD7 is restricted primarily to granule cells; therefore, there should be minimal contribution of CHD7 protein from other cell types. The Atoh1-Cre transgenic line preferentially mediates knockout of Chd7 from granule cells in the anterior part of the cerebellum. While isolation of the anterior cerebellum was performed to enrich for granule cells with deletion of Chd7, a small proportion of interspersed granule cells without Chd7 deletion will ultimately be included in the sample. Therefore, we attribute the CHD7-binding signal in Chd7-conditional knockout mouse cerebellum to CHD7-occupied sites in the minimal population of granule cells that do not express the Atoh1-Cre transgene. Thus, while the majority of cells in our ChIP sample are granule neurons with deletion of Chd7, minimal signal is detectable from granule cells without transgene expression. In addition, we cannot rule out the possibility that low levels of CHD7 expression in non-granule cell types may also contribute to the signal detected by ChIP-seq.

### RNA-seq analysis

Differential mRNA-seq analysis was performed for RNA micro-dissected from the EGL of cerebellum of P4 control and conditional knockout mice. RNA-seq alignment was performed using STAR (v2.5.2b). Differential gene expression analysis was performed using EdgeR (v3.24.1) and DESeq2 (v1.18.1). Only genes found to be significantly dysregulated by both EdgeR and DESeq2 were considered significantly changed.

### ATAC-seq analysis

Paired-end reads of 50 base pairs were obtained for all datasets. Samples were aligned to the mm10 genome using Bowtie2 with default parameters for Galaxy platform. Reads were then filtered for a map quality score greater than 10 (mapQuality > 10). Peaks were called using MACS2 on individual replicates. All peaks identified in at least two control or conditional knockout samples were used in downstream analyses.

### Hi-C analysis

#### Loop and contact domain analyses

Loops were identified in independently in CHD7 control and conditional knockout mice from Knight-Ruiz-normalized contact matrices using the juicer (v1.19.02) HiCCUPS algorithm at 10 kb on a CPU (–cpu) with default parameters^46^. Differential loops were identified between CHD7 control and conditional knockout contact matrices using juicer (v1.19.02) HiCCUPSDiff. CHD7 control and conditional knockout loops were merged if the left anchor of the control loop was within 10 kb of the left anchor of the conditional knockout loop and if the right anchor of the control loop was within 10 kb of the right anchor of the conditional knockout loop. This merging was performed through pgltools (Greenwald et al.^74^) Enhancer–promoter loops were defined as loops that overlapped an enhancer in one anchor and a promoter in the paired anchor.

Contact domains were identified independently in CHD7 control and conditional knockout mice by running the juicer-tools (v.1.19.02) Arrowhead algorithm on Knight-Ruiz-normalized contact matrices^46^. CHD7 control and conditional knockout contact domain lists were combined for downstream analyses. Contact domains were merged if their domain borders were within 20 kb of each other. If the left border of a contact domain was within 25 kb of a loop’s left anchor and the right border of the contact domain was within 25 kb of a loop’s right anchor, the contact domain was considered a loop domain. Compartmental domains were defined as contact domains that were not identified as loop domains. Change in intradomain contact frequency was quantified by log2-transforming the ratio of number of observed/expected contacts in CHD7-conditional knockout contact matrices to the number of observed/expected contacts in CHD7 control contact matrices within the same domain. Domain-level chromatin accessibility or H3K27ac ChIP-seq signal was analyzed by log2-transforming the average fold-change of signal at ATAC-seq or H3K27ac ChIP-seq peaks within a contact domain.

#### Compartmentalization analyses

Eigenvectors for each chromosome were independently calculated from the pooled sets of Chd7 control and Chd7 cKO KR-normalized contact matrices using juicer-tools (v1.19.02) eigenvector at 150 kb resolution. A/B compartments were identified from the eigenvectors using the number of H3K4me3 peaks (called in Chd7 control) overlapping the 150 kb bins as previously described (Norrie et al.^75^). Eigenvector signs were oriented so that positive value bins were correlated with a higher number of H3K4me3 peaks and labeled as the euchromatin-associated A compartment. The negative value bins were labeled as the heterochromatin-associated B compartment. A density plot was made showing high genome-wide correlation between Chd7 control and Chd7 cKO eigenvectors in 150 kb bins.

### Gene ontology analysis

ChIP-seq peaks were used to assess significantly enriched mouse phenotypes using GREAT analysis tool. Analyses were completed using the whole genome as background and single nearest gene associations within 100 kb. For enriched biological processes and pathways analysis, PANTHER Classification System (v15.0) was used. ENSEMBL gene IDs were input into PANTHER analysis tool and all expressed genes were used as background. Fisher’s exact test followed by Bonferroni multiple testing correction was used to identify significantly enriched terms.

#### Human-mouse intersectional analysis

All P4 expressed CHD7-regulated genes were considered for further analyses. To ensure no bias due to conversion of mouse genes to human genes, Ensembl genes without mouse and human IDs were filtered out. Single nearest human genes within 100 kb of a human CHD7 ChIP-seq peak were identified and considered human CHD7-regulated genes. Hypergeometric analysis of the overlap between CHD7 mouse and human target genes was performed.

### Ethics approval

Mice were maintained in a pathogen-free environment. All procedures involving animals were performed according to protocols approved by the Animal Studies Committee of Washington University School of Medicine and in accordance with both the National Institute of Health Standings Committee on Animals as well as the National Institutes of Health guidelines. The project was reviewed and approved by the Washington University Human Research Protection Office (HRPO). It was determined not to involve activities subject to Institutional Review Board oversight because all data and specimens are from deceased individuals, and therefore the requirement for informed consent was waived by the HRPO. The study was conducted in accordance to the criteria set by the Declaration of Helsinki.

## Supplementary information

Supplementary Information

Description of Additional Supplementary Files

Supplementary Data 1

## Data Availability

The data that support this study are available from the corresponding authors upon reasonable request. The RNA-seq, ChIP-seq, ATAC-seq, and Hi-C sequencing data generated in this study have been deposited in the Gene Expression Omnibus (GEO) database under accession code GSE164360. The human HiC TADs were visualized using the Yue Lab 3D Genome Browser and data used was acquired from GEO under accession GSE87112. Source data are provided with this paper. Code used to generate figures is available upon request. Source data are provided with this paper.

## Source data

Source Data

## References

[1] Ronan JL, Wu W, Crabtree GR (2013). From neural development to cognition: unexpected roles for chromatin. Nat. Rev. Genet..

[2] Gallegos DA, Chan U, Chen LF, West AE (2018). Chromatin regulation of neuronal maturation and plasticity. Trends Neurosci..

[3] Yamada T (2014). Promoter decommissioning by the NuRD chromatin remodeling complex triggers synaptic connectivity in the mammalian brain. Neuron.

[4] Nitarska J (2016). A functional switch of nurd chromatin remodeling complex subunits regulates mouse cortical development. Cell Rep..

[5] Goodman JV, Bonni A (2019). Regulation of neuronal connectivity in the mammalian brain by chromatin remodeling. Curr. Opin. Neurobiol..

[6] O’Roak BJ (2014). Recurrent de novo mutations implicate novel genes underlying simplex autism risk. Nat. Commun..

[7] Bernier R (2014). Disruptive CHD8 mutations define a subtype of autism early in development. Cell.

[8] Weiss K (2016). De novo mutations in CHD4, an ATP-dependent chromatin remodeler gene, cause an intellectual disability syndrome with distinctive dysmorphisms. Am. J. Hum. Genet..

[9] Pilarowski GO (2018). Missense variants in the chromatin remodeler CHD1 are associated with neurodevelopmental disability. J. Med. Genet..

[10] Vissers LE (2004). Mutations in a new member of the chromodomain gene family cause CHARGE syndrome. Nat. Genet..

[11] Bergman JE (2011). CHD7 mutations and CHARGE syndrome: the clinical implications of an expanding phenotype. J. Med. Genet..

[12] Legendre M (2012). Antenatal spectrum of CHARGE syndrome in 40 fetuses with CHD7 mutations. J. Med. Genet..

[13] Yu T (2013). Deregulated FGF and homeotic gene expression underlies cerebellar vermis hypoplasia in CHARGE syndrome. Elife.

[14] Legendre M (2017). Phenotype and genotype analysis of a French cohort of 119 patients with CHARGE syndrome. Am. J. Med. Genet. Part C., Semin. Med. Genet..

[15] Whittaker DE (2017). The chromatin remodeling factor CHD7 controls cerebellar development by regulating reelin expression. J. Clin. Invest..

[16] Feng W (2017). Chd7 is indispensable for mammalian brain development through activation of a neuronal differentiation programme. Nat. Commun..

[17] Goodman JV (2020). The chromatin remodeling enzyme Chd4 regulates genome architecture in the mouse brain. Nat. Commun..

[18] Ramon y Cajal S (1911). Histologie du système nerveux de l’homme et des vertébrés. Maloine, Paris.

[19] Martinez S, Andreu A, Mecklenburg N, Echevarria D (2013). Cellular and molecular basis of cerebellar development. Front. Neuroanat..

[20] Altman, J. & Bayer S. A. *Development of the Cerebellar System* (Wiley, 1997).

[21] Chang JC (2015). Mitotic events in cerebellar granule progenitor cells that expand cerebellar surface area are critical for normal cerebellar cortical lamination in mice. J. Neuropathol. Exp. Neurol..

[22] Legue E (2016). Differential timing of granule cell production during cerebellum development underlies generation of the foliation pattern. Neural Dev..

[23] Feng W, Shao C, Liu HK (2017). Versatile Roles of the Chromatin Remodeler CHD7 during Brain Development and Disease. Front. Mol. Neurosci..

[24] Andersson R, Sandelin A (2020). Determinants of enhancer and promoter activities of regulatory elements. Nat. Rev. Genet.

[25] Creyghton MP (2010). Histone H3K27ac separates active from poised enhancers and predicts developmental state. Proc. Natl Acad. Sci. USA.

[26] Zentner GE, Tesar PJ, Scacheri PC (2011). Epigenetic signatures distinguish multiple classes of enhancers with distinct cellular functions. Genome Res..

[27] Rada-Iglesias A (2011). A unique chromatin signature uncovers early developmental enhancers in humans. Nature.

[28] Zaret KS, Carroll JS (2011). Pioneer transcription factors: establishing competence for gene expression. Genes Dev..

[29] Buecker C (2014). Reorganization of enhancer patterns in transition from naive to primed pluripotency. Cell Stem Cell.

[30] Bonn S (2012). Tissue-specific analysis of chromatin state identifies temporal signatures of enhancer activity during embryonic development. Nat. Genet..

[31] Legue E, Riedel E, Joyner AL (2015). Clonal analysis reveals granule cell behaviors and compartmentalization that determine the folded morphology of the cerebellum. Development.

[32] Lejeune E, Dortdivanlioglu B, Kuhl E, Linder C (2019). Understanding the mechanical link between oriented cell division and cerebellar morphogenesis. Soft Matter.

[33] Lawton, A. K. et al. Cerebellar folding is initiated by mechanical constraints on a fluid-like layer without a cellular pre-pattern. *Elife* **8**, 10.7554/eLife.45019 (2019). 10.7554/eLife.45019 PMC6467563 30990415

[34] Becker EB, Stoodley CJ (2013). Autism spectrum disorder and the cerebellum. Int. Rev. Neurobiol..

[35] Laidi C (2017). Cerebellar anatomical alterations and attention to eyes in autism. Sci. Rep..

[36] Stoodley CJ (2017). Altered cerebellar connectivity in autism and cerebellar-mediated rescue of autism-related behaviors in mice. Nat. Neurosci..

[37] Cederquist GY (2012). An inherited TUBB2B mutation alters a kinesin-binding site and causes polymicrogyria, CFEOM and axon dysinnervation. Hum. Mol. Genet..

[38] Heinzen EL (2018). De novo and inherited private variants in MAP1B in periventricular nodular heterotopia. PLoS Genet..

[39] Creyghton MP (2008). H2AZ is enriched at polycomb complex target genes in ES cells and is necessary for lineage commitment. Cell.

[40] John S (2011). Chromatin accessibility pre-determines glucocorticoid receptor binding patterns. Nat. Genet..

[41] Schnetz MP (2009). Genomic distribution of CHD7 on chromatin tracks H3K4 methylation patterns. Genome Res.

[42] Schnetz, M. P. et al. CHD7 targets active gene enhancer elements to modulate ES cell-specific gene expression. *PLoS Genet*. **6** (2010). 10.1371/journal.pgen.1001023 PMC2904778 20657823

[43] Ray J (2019). Chromatin conformation remains stable upon extensive transcriptional changes driven by heat shock. Proc. Natl Acad. Sci. USA.

[44] Schoenfelder S, Fraser P (2019). Long-range enhancer-promoter contacts in gene expression control. Nat. Rev. Genet.

[45] Dixon JR (2012). Topological domains in mammalian genomes identified by analysis of chromatin interactions. Nature.

[46] Rao SS (2014). A 3D map of the human genome at kilobase resolution reveals principles of chromatin looping. Cell.

[47] Rowley MJ, Corces VG (2018). Organizational principles of 3D genome architecture. Nat. Rev. Genet.

[48] Oudelaar AM (2020). Dynamics of the 4D genome during in vivo lineage specification and differentiation. Nat. Commun..

[49] Clemens AW (2020). MeCP2 represses enhancers through chromosome topology-associated DNA methylation. Mol. Cell.

[50] Boxer LD (2020). MeCP2 represses the rate of transcriptional initiation of highly methylated long genes. Mol. Cell.

[51] Bayly PV, Okamoto RJ, Xu G, Shi Y, Taber LA (2013). A cortical folding model incorporating stress-dependent growth explains gyral wavelengths and stress patterns in the developing brain. Phys. Biol..

[52] Kroenke CD, Bayly PV (2018). How forces fold the cerebral cortex. J. Neurosci..

[53] Borrell V (2018). How cells fold the cerebral cortex. J. Neurosci..

[54] Whittaker, D. E. et al. Distinct cerebellar foliation anomalies in a CHD7 haploinsufficient mouse model of CHARGE syndrome. *Am. J. Med. Genet. Part C, Semin. Med. Genet.* **175**, 10.1002/ajmg.c.31595 (2017). 10.1002/ajmg.c.31595 PMC5765394 29168327

[55] Basson MA (2014). Epistatic interactions between Chd7 and Fgf8 during cerebellar development: Implications for CHARGE syndrome. Rare Dis. (Austin, Tex.).

[56] Donovan APA (2017). Cerebellar vermis and midbrain hypoplasia upon conditional deletion of Chd7 from the embryonic mid-hindbrain Region. Front. Neuroanat..

[57] Sudarov A, Joyner AL (2007). Cerebellum morphogenesis: the foliation pattern is orchestrated by multi-cellular anchoring centers. Neural Dev..

[58] Wang SS, Kloth AD, Badura A (2014). The cerebellum, sensitive periods, and autism. Neuron.

[59] Feng W (2013). The chromatin remodeler CHD7 regulates adult neurogenesis via activation of SoxC transcription factors. Cell Stem Cell.

[60] Matei V (2005). Smaller inner ear sensory epithelia in Neurog 1 null mice are related to earlier hair cell cycle exit. Dev Dyn..

[61] Heiney SA, Wohl MP, Chettih SN, Ruffolo LI, Medina JF (2014). Cerebellar-dependent expression of motor learning during eyeblink conditioning in head-fixed mice. J. Neurosci..

[62] Yang Y (2016). Chromatin remodeling inactivates activity genes and regulates neural coding. Science.

[63] Valnegri P (2017). RNF8/UBC13 ubiquitin signaling suppresses synapse formation in the mammalian brain. Nat. Commun..

[64] Puram SV (2011). A TRPC5-regulated calcium signaling pathway controls dendrite patterning in the mammalian brain. Genes Dev..

[65] Amende I (2005). Gait dynamics in mouse models of Parkinson’s disease and Huntington’s disease. J. Neuroeng. Rehab..

[66] Konishi Y, Stegmuller J, Matsuda T, Bonni S, Bonni A (2004). Cdh1-APC controls axonal growth and patterning in the mammalian brain. Science.

[67] Kim AH (2009). A centrosomal Cdc20-APC pathway controls dendrite morphogenesis in postmitotic neurons. Cell.

[68] Yang Y (2009). A Cdc20-APC ubiquitin signaling pathway regulates presynaptic differentiation. Science.

[69] Chen X (2019). The transcriptional regulator snon promotes the proliferation of cerebellar granule neuron precursors in the postnatal mouse brain. J. Neurosci..

[70] Majidi SP (2019). Chromatin environment and cellular context specify compensatory activity of paralogous MEF2 transcription factors. Cell Rep..

[71] Lieberman-Aiden E (2009). Comprehensive mapping of long-range interactions reveals folding principles of the human genome. Science.

[72] Jongmans MC (2006). CHARGE syndrome: the phenotypic spectrum of mutations in the CHD7 gene. J. Med. Genet..

[73] Janssen N (2012). Mutation update on the CHD7 gene involved in CHARGE syndrome. Hum. Mutat..

[74] Greenwald WW (2017). Pgltools: a genomic arithmetic tool suite for manipulation of Hi-C peak and other chromatin interaction data. BMC Bioinformatics.

[75] Norrie JL (2019). Nucleome Dynamics during Retinal Development.. Neuron.

