# Supplementary material for: CHARGE syndrome protein CHD7 regulates epigenomic activation of enhancers in granule cell precursors and gyrification of the cerebellum
Source: Nat Commun. 2021 Sep 29;12:5702. doi: 10.1038/s41467-021-25846-3 (PMC8481233; doi:10.1038/s41467-021-25846-3)
Supplement: Supplementary file 1 — Supplementary Information [file 41467_2021_25846_MOESM1_ESM.pdf]

Supplementary Information

CHARGE Syndrome Protein CHD7 Regulates Epigenomic Activation of Enhancers in Granule Cell Precursors and Gyrification of the Cerebellum

Naveen C. Reddy, Shahriyar P. Majidi, Lingchun Kong, Mati Nemera, Cole J. Ferguson, Michael Moore, Tassia M. Goncalves, Hai-Kun Liu, James A. J. Fitzpatrick, Guoyan Zhao, Tomoko Yamada, Azad Bonni, Harrison W. Gabel

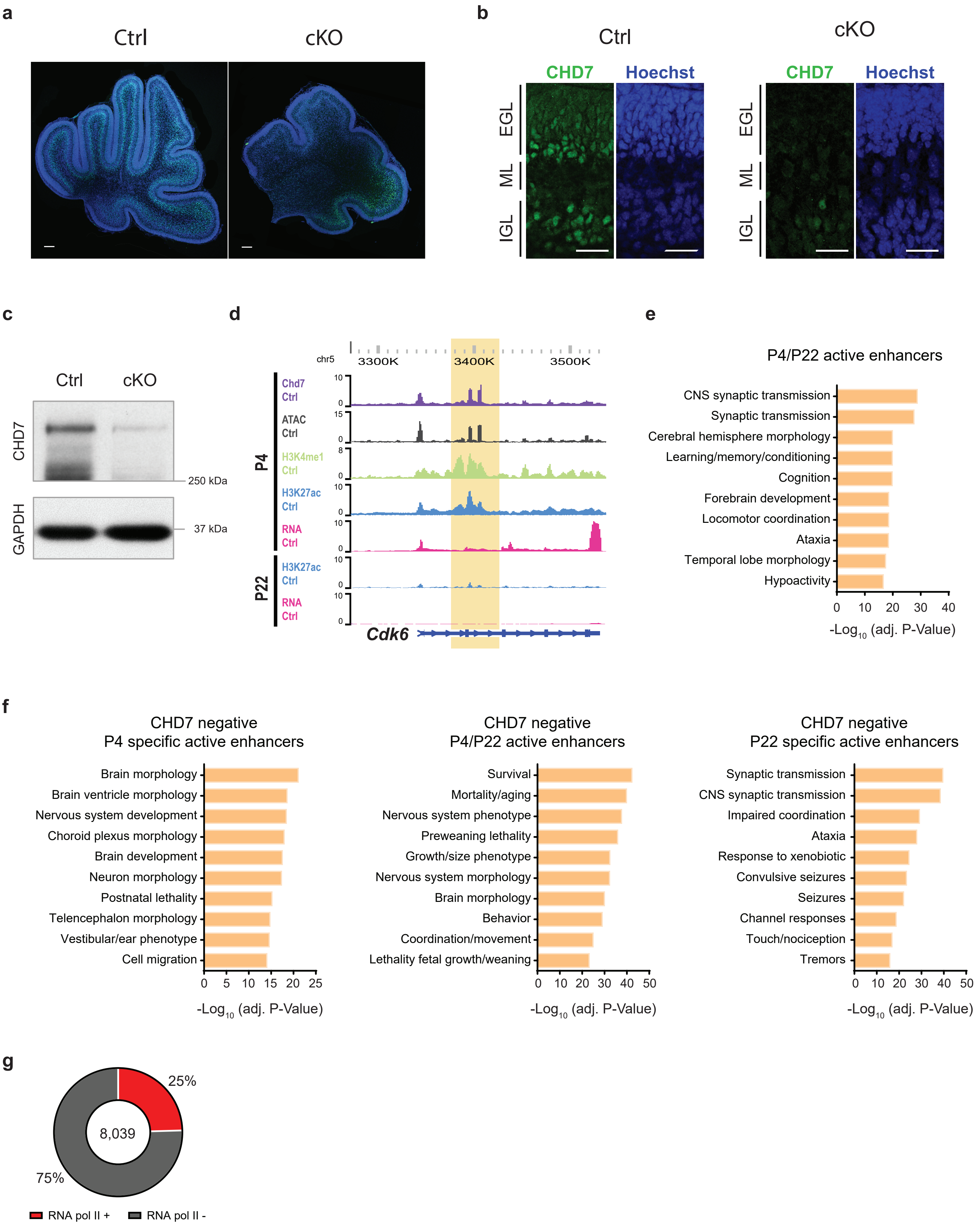
a
Ctrl
cKO
b
Ctrl
cKO
CHD7
Hoechst
EGL
ML
IGL
c
Ctrl
cKO
CHD7
GAPDH
250 kDa
37 kDa
d
chr5
3300K
3400K
3500K
P4
Chd7 Ctrl
ATAC Ctrl
H3K4me1 Ctrl
H3K27ac Ctrl
RNA Ctrl
P22
H3K27ac Ctrl
RNA Ctrl
Cdk6
e
P4/P22 active enhancers
CNS synaptic transmission
Synaptic transmission
Cerebral hemisphere morphology
Learning/memory/conditioning
Cognition
Forebrain development
Locomotor coordination
Ataxia
Temporal lobe morphology
Hypoactivity
0 10 20 30 40
-Log10 (adj. P-Value)
f
CHD7 negative P4 specific active enhancers
Brain morphology
Brain ventricle morphology
Nervous system development
Choroid plexus morphology
Brain development
Neuron morphology
Postnatal lethality
Telencephalon morphology
Vestibular/ear phenotype
Cell migration
0 5 10 15 20 25
-Log10 (adj. P-Value)
CHD7 negative P4/P22 active enhancers
Survival
Mortality/aging
Nervous system phenotype
Preweaning lethality
Growth/size phenotype
Nervous system morphology
Brain morphology
Behavior
Coordination/movement
Lethality fetal growth/weaning
0 10 20 30 40 50
-Log10 (adj. P-Value)
CHD7 negative P22 specific active enhancers
Synaptic transmission
CNS synaptic transmission
Impaired coordination
Ataxia
Response to xenobiotic
Convulsive seizures
Seizures
Channel responses
Touch/nociception
Tremors
0 10 20 30 40 50
-Log10 (adj. P-Value)
g
25%
8,039
75%
RNA pol II +
RNA pol II -

Supplementary Fig. 1: Conditional CHD7 knockout in mouse cerebellum.

a. P4 sagittal cerebellar sections from CHD7 ctrl (left) and cKO (right) mouse lines were subjected to immunohistochemistry by using antibodies recognizing CHD7 and DNA dye bisbenzimide (Hoechst). Experiment was repeated two times for each condition. Scalebar: 100um.

b. P4 cerebellar sections from CHD7 ctrl (left) and cKO (right) mouse lines were subjected to immunohistochemistry by using antibodies recognizing CHD7 (left) and DNA dye bisbenzimide (Hoechst, right). EGL, external granule layer; ML, molecular layer; IGL, internal granule layer. Experiment was repeated two times for each condition. Scalebar: 25um.

c. Immunoblotting of CHD7 (top) and GAPDH (bottom) in lysates of P4 mouse cerebellum harvested from CHD7 ctrl and cKO mice. Experiment was repeated three times for each condition.

d. WashU Epigenome Browser view of a CHD7 bound P4-specific active enhancer (highlighted). ChIP-seq coverage for CHD7, H3K27ac, H3K4me1, as well as, ATAC-seq and RNA-seq is shown.

e. GREAT analysis showing enriched mouse phenotypes associated with enhancers active at P4 and P22.

f. GREAT analysis showing enriched mouse phenotypes associated with CHD7-negative enhancers active at P4 specifically (left), P4 and P22 (middle), and P22 specifically (right).

g. Pie chart representing proportion of CHD7 occupied poised enhancers displaying RNApolII binding.

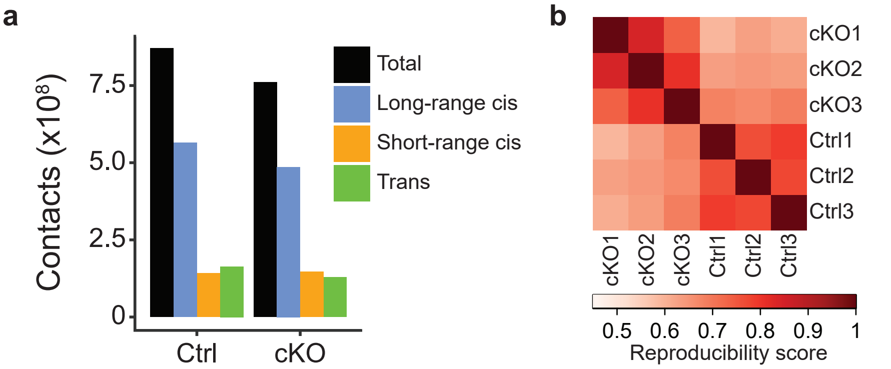

c

	Ctrl	cKO	Total
TADs	2,311	2,306	2,373
Loops	9,764	11,422	13,559
Loop domains	588	589	602
Comp. domains	1,723	1,717	1,771

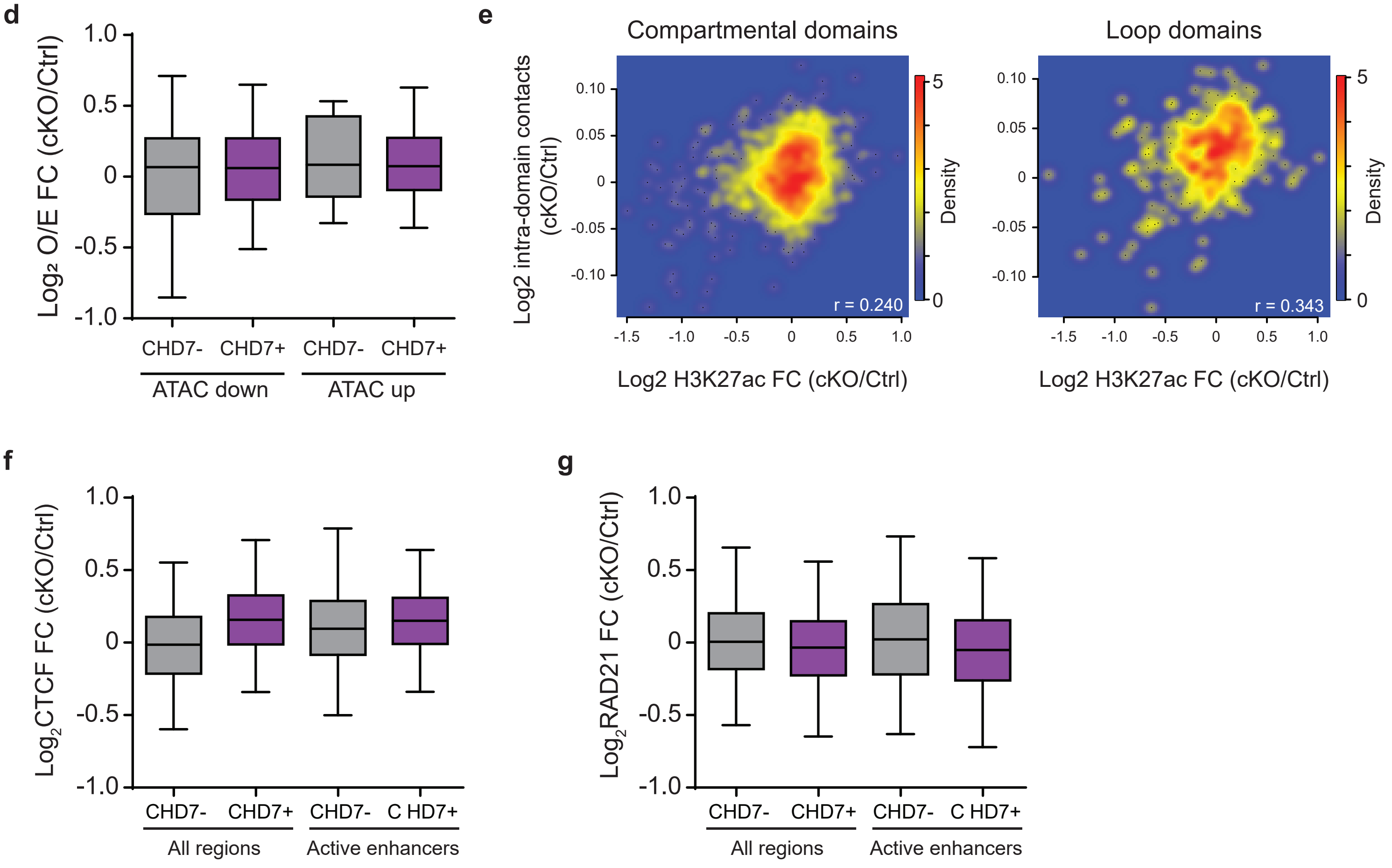

Supplementary Fig 2: Conditional CHD7 knockout minimally alters genomic architecture and associated regulatory factors.

a. Distribution of pooled Hi-C contacts in CHD7 ctrl and cKO cerebellum.
b. Hi-C Spector analysis for reproducibility of CHD7 ctrl and cKO biological replicates.
c. Table summarizing results of Hi-C analysis.
d. Box-whisker plots showing the median, 1st quartile, 3rd quartile, and whiskers displaying the 5th and 95th percentiles of intradomain loops representing median and distribution of the log2 fold change of observed over expected Hi-C signal at CHD7 bound and unoccupied domains separated by decreasing (n=59, n=716) or increasing (n=14, n=192) ATAC-seq signal change in CHD7 cKO over ctrl.
e. Density plots comparing the log2 fold change in H3K27ac-seq signal at CHD7 bound peaks in a contact domain with the log2 fold change in Hi-C contacts in compartmental domains (left) and loop domains (right).
f. Box-whisker plots showing the median, 1st quartile, 3rd quartile, and whiskers displaying the 5th and 95th percentiles of CTCF occupied regions representing median and distribution of the log2 fold change of CTCF signal at all regions (n=13,758, n=1,088) and active enhancer (n=598, n=606).
g. Box-whisker plots showing the median, 1st quartile, 3rd quartile, and whiskers displaying the 5th and 95th percentiles of RAD21 occupied regions representing median and distribution of the log2 fold change of RAD21 signal at all regions (n=15,188, n=8.623) and active enhancer (n=1,209, n=2,506).

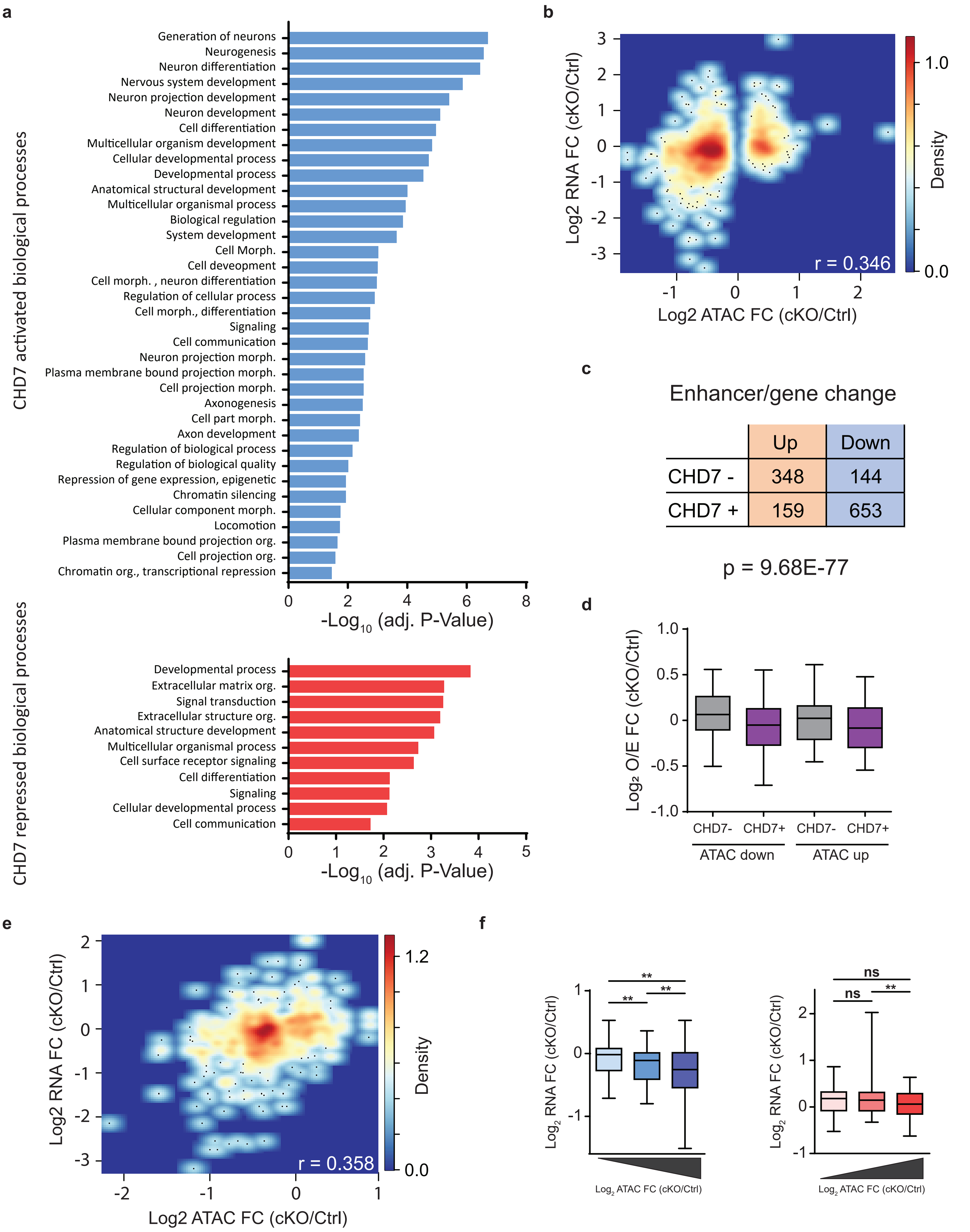
a
CHD7 activated biological processes
Generation of neurons
Neurogenesis
Neuron differentiation
Nervous system development
Neuron projection development
Neuron development
Cell differentiation
Multicellular organism development
Cellular developmental process
Developmental process
Anatomical structural development
Multicellular organismal process
Biological regulation
System development
Cell Morph.
Cell deveopment
Cell morph. , neuron differentiation
Regulation of cellular process
Cell morph., differentiation
Signaling
Cell communication
Neuron projection morph.
Plasma membrane bound projection morph.
Cell projection morph.
Axonogenesis
Cell part morph.
Axon development
Regulation of biological process
Regulation of biological quality
Repression of gene expression, epigenetic
Chromatin silencing
Cellular component morph.
Locomotion
Plasma membrane bound projection org.
Cell projection org.
Chromatin org., transcriptional repression
0 2 4 6 8
-Log10 (adj. P-Value)
CHD7 repressed biological processes
Developmental process
Extracellular matrix org.
Signal transduction
Extracellular structure org.
Anatomical structure development
Multicellular organismal process
Cell surface receptor signaling
Cell differentiation
Signaling
Cellular developmental process
Cell communication
0 1 2 3 4 5
-Log10 (adj. P-Value)
b
Log2 RNA FC (cKO/Ctrl)
Log2 ATAC FC (cKO/Ctrl)
r = 0.346
Density
1.0
0.0
c
Enhancer/gene change
Up Down
CHD7 - 348 144
CHD7 + 159 653
p = 9.68E-77
d
Log2 O/E FC (cKO/Ctrl)
CHD7- CHD7+ CHD7- CHD7+
ATAC down
ATAC up
e
Log2 RNA FC (cKO/Ctrl)
Log2 ATAC FC (cKO/Ctrl)
r = 0.358
Density
1.2
0.0
f
Log2 RNA FC (cKO/Ctrl)
**
**
**
Log2 ATAC FC (cKO/Ctrl)
ns
ns
**
Log2 ATAC FC (cKO/Ctrl)

Supplementary Fig. 3: Conditional CHD7 knockout disrupts many biological processes by changing enhancer accessibility and corresponding gene expression.

a. Panther gene ontology analysis of significantly dysregulated genes associated with CHD7 ChIP-seq peaks ($p <= 0.05$). Significantly enriched biological processes are shown for CHD7 activated (top, blue) and CHD7 repressed (bottom, red) genes.
b. Density plots comparing the log2 fold change in enhancer ATAC-seq signal to the log2 fold change in RNA expression of correspond gene.
c. Table displaying number of significantly changed enhancers in the CHD7 cKO for which transcript levels of the gene linked to that enhancer change in the same direction as the enhancer. Enhancers are paired to a gene within 100Kb and withing the same TAD. P-value = 9.68E-77, one-sided Chi-squared test.
d. Box plots showing the median, 1st quartile, 3rd quartile, and whiskers displaying the 5th and 95th percentiles of E-P pairs representing median and distribution of the log2 fold change of observed over expected Hi-C signal at CHD7 bound (2,203) and unoccupied (2,972) domains separated by decreasing (n=116, n=558) or increasing (n=308, n=156) ATAC-seq signal change in CHD7 cKO over ctrl.
e. Density plots comparing the log2 fold change in domain ATAC-seq signal to the log2 fold change in RNA expression of correspond genes.
f. Box-whisker plots showing the median, 1st quartile, 3rd quartile, and whiskers displaying the 5th and 95th percentiles of CHD7 bound proximal genes within TADs representing median and distribution of the log2 fold change of RNA signal in CHD7 cKO over CHD7 control. Active enhancers were divided into three groups based on significantly increasing or decreasing changes in ATAC-seq signal. We then quantified the change in

gene expression of corresponding genes within groups defined by ATAC-seq changes. Boxplots are divided into groups of decreasing (left; n=399, n=399, n=398) and increasing (right; n=113, n=113, n=112) ATAC-seq fold change **p < 0.01, unpaired two-sided t-test, Tukey's multiple comparison test.

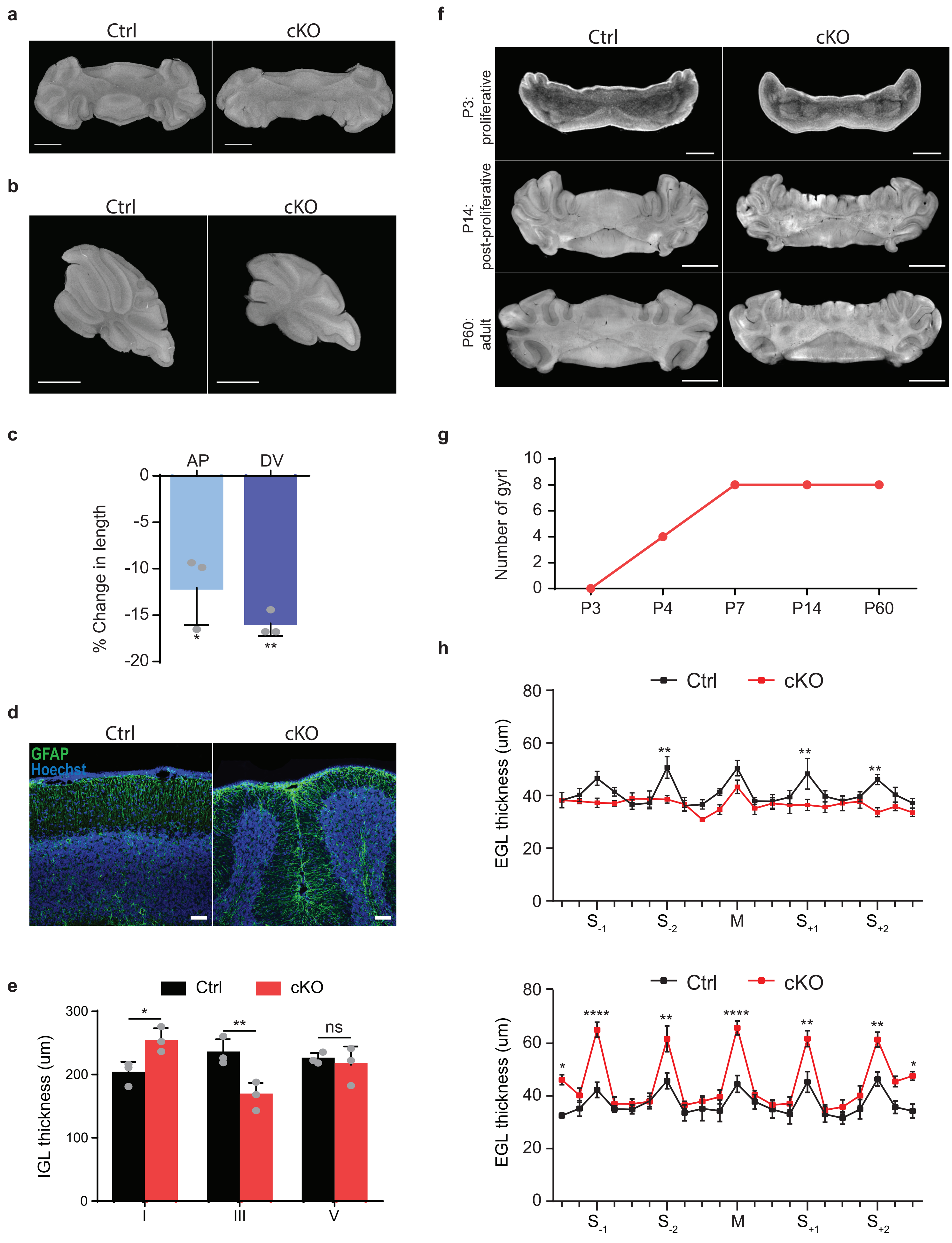

a
Ctrl
cKO
b
Ctrl
cKO
c
AP
DV
% Change in length
0
-5
-10
-15
-20
*
**
d
Ctrl
cKO
GFAP
Hoechst
e
Ctrl
cKO
IGL thickness (um)
300
200
100
0
*
**
ns
I
III
V
f
Ctrl
cKO
P3: proliferative
P14: post-proliferative
P60: adult
g
Number of gyri
10
8
6
4
2
0
P3
P4
P7
P14
P60
h
Ctrl
cKO
EGL thickness (um)
80
60
40
20
0
**
**
**
S-1
S-2
M
S+1
S+2
Ctrl
cKO
EGL thickness (um)
*
****
**
****
**
**
*
S-1
S-2
M
S+1
S+2

Supplementary Fig. 4: Cerebellar microgyria appears at the proliferative stage of granule cell precursor development.

a. Nano CT mediolateral scans of lobule IX of Ctrl (left) and cKO (right) p56 mouse cerebella. Scale bar: 2mm.

b. Nano CT midline sagittal scans of Ctrl (left) and cKO (right) p56 mouse cerebella. Scale bar: 2mm.

c. Bar graphs showing % change in total cerebellar (Cb) anterior-posterior (AP) and dorsoventral (DV) measurement upon CHD7 cKO in p56 mice (n=3 for each condition). Length is shown as mean □ s.e.m for each measurement. *p<0.05, **p<0.01 Paired two-sided t-test.

d. Immunohistochemistry analysis of Ctrl and cKO p56 mice with antibodies recognizing GFAP, a marker of glial cells, and Hoechst. Experiment was repeated two times for each condition. Scalebar: 100um.

e. Bar graph showing internal granule cell layer (IGL) thickness +/- s.e.m. of lobules I, III, and V for Ctrl and cKO p56 mice (n=3 for each condition). p= 0.0382 (I), p=0.0072 (III), p=0.9556 (V) Two-way ANOVA test; ns, not significant.

f. Nano CT scans were taken of multiple stages of granule cell development. Onset of postnatal GCP proliferation (P3: proliferative); post GCP proliferation (P14: post-proliferative); adult granule cells (P60: adult). Scale bar: 500um (top), 2mm (middle and bottom).

g. Graph displaying the time course of formation of additional gyri in CHD7 cKO mice.

h. Graphs displaying the thickness of the EGL +/- s.e.m. along the mediolateral plane of Ctrl and cKO P3 (top) and P4 (bottom) mice. Midline (M), Sulcus (S). *$p<0.05$, **$p<0.01$, ****$p<0.0001$.Two-way ANOVA.

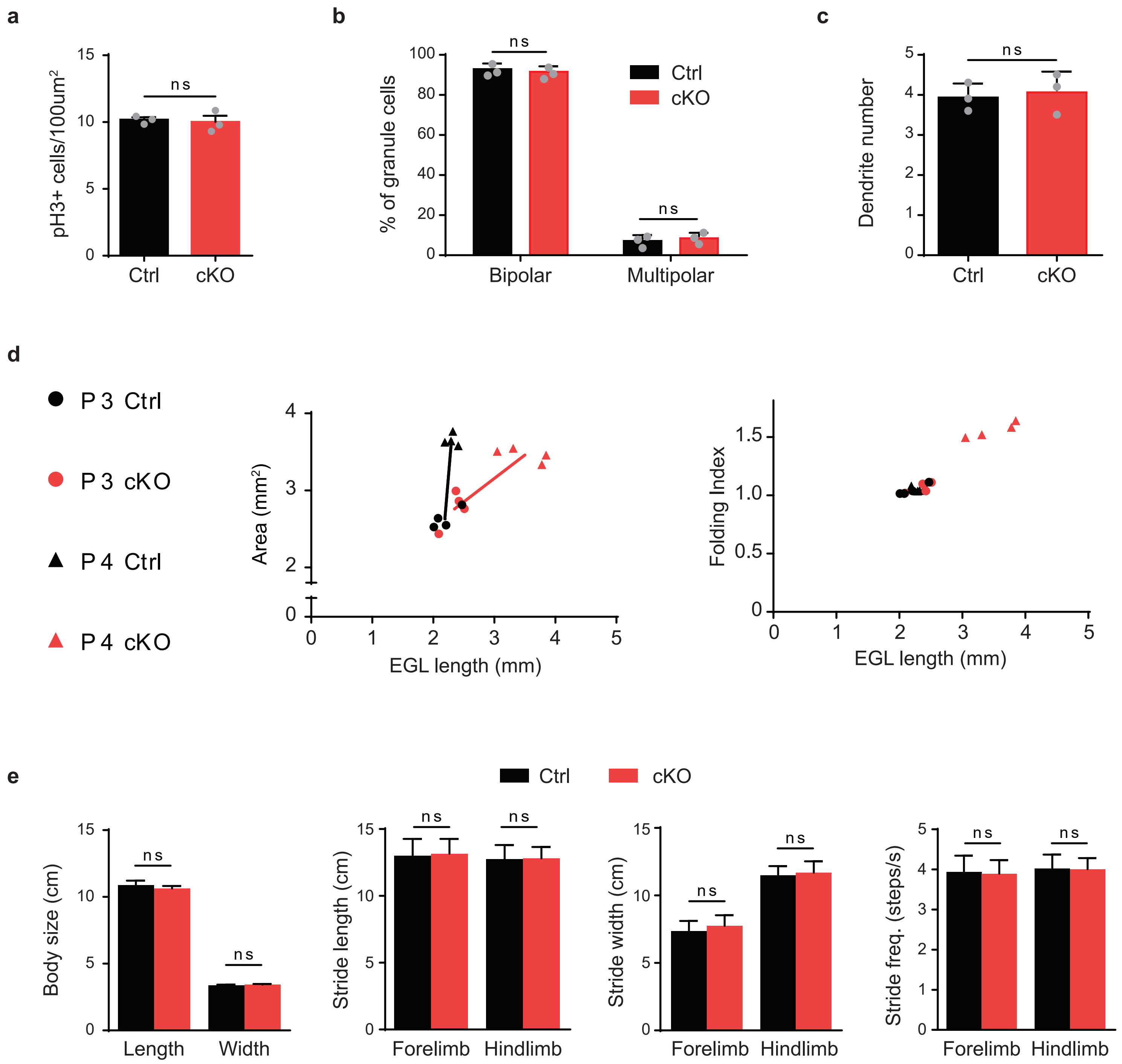

a
pH3+ cells/100um²
15
10
5
0
ns
Ctrl
cKO
b
% of granule cells
100
80
60
40
20
0
ns
ns
Ctrl
cKO
Bipolar
Multipolar
c
Dendrite number
5
4
3
2
1
0
ns
Ctrl
cKO
d
P3 Ctrl
P3 cKO
P4 Ctrl
P4 cKO
Area (mm²)
4
3
2
0
EGL length (mm)
0
1
2
3
4
5
Folding Index
1.5
1.0
0.5
0
EGL length (mm)
e
Ctrl
cKO
Body size (cm)
15
10
5
0
ns
ns
Length
Width
Stride length (cm)
ns
ns
Forelimb
Hindlimb
Stride width (cm)
ns
ns
Forelimb
Hindlimb
Stride freq. (steps/s)
ns
ns
Forelimb
Hindlimb

Supplementary Fig. 5: Loss of CHD7 results in mediolateral differential expansion of granule cell precursors without perturbing post-proliferative granule cell development and gross motor output of mouse cerebellum.

a. Immunohistochemistry analysis of posterior cerebellum of Ctrl and cKO mice (n=3 for each condition) with antibodies recognizing phosphorylated histone H3 (pH3), a marker of dividing cells. Dividing cells were counted in the region posterior to the secondary fissure of the cerebellum (lobules IX and X), referred to in shorthand as the posterior cerebellum. Bar graphs show mean pH3 positive cells per $100um^2$ +/- s.e.m. ns = non-significant. Paired two-sided t-test.
b. CHD7 Ctrl (n=3) and cKO (n=3) mice were electroporated at P6 with the pCAG-GFP vector and fixed 2 days later in order to identify migrating granule cells. Granule cells with a parallel fiber and one leading process were considered to be bipolar, while granule cells with a parallel fiber and multiple processes were considered to be multipolar. Percentage of bipolar and multipolar granule cells +/- s.e.m. was calculated for each mouse and analyzed for significance. One-sided Chi-square test. ns, not significant.
c. CHD7 Ctrl (n=3) and cKO (n=3) mice were electroporated at P6 with the pCAG-GFP vector and fixed 8 days later in order to identify mature granule cells. The number of dendrites of GFP positive cells were counted in each mouse and then averaged. Bar graphs show average dendrite numbers +/- s.e.m. . Two-sided t-test. ns, not significant.
d. Scatter plots of cross sectional P3 and P4 cerebellum area (left) and folding index (right) vs EGL length. EGL length was measured in the anterior cerebellar vermis using Nano CT sections. Area was also quantified using Nano CT sections of the anterior cerebellar vermis. Folding index is the ratio of the EGL length to the length of the anterior

cerebellar vermis. Slope of Ctrl (black line) and cKO (red line) define expansion properties of cerebellum.

e. Analysis of body size and gait dynamics by Digigait assay did not reveal deficits in sex-matched cKO mice (n=14) relative to control (n=11) littermates on stride-related variables for both the forelimbs and hindlimbs. Data are presented as mean values +/- s.e.m. Two-way ANOVA with Sidak's multiple comparison test.

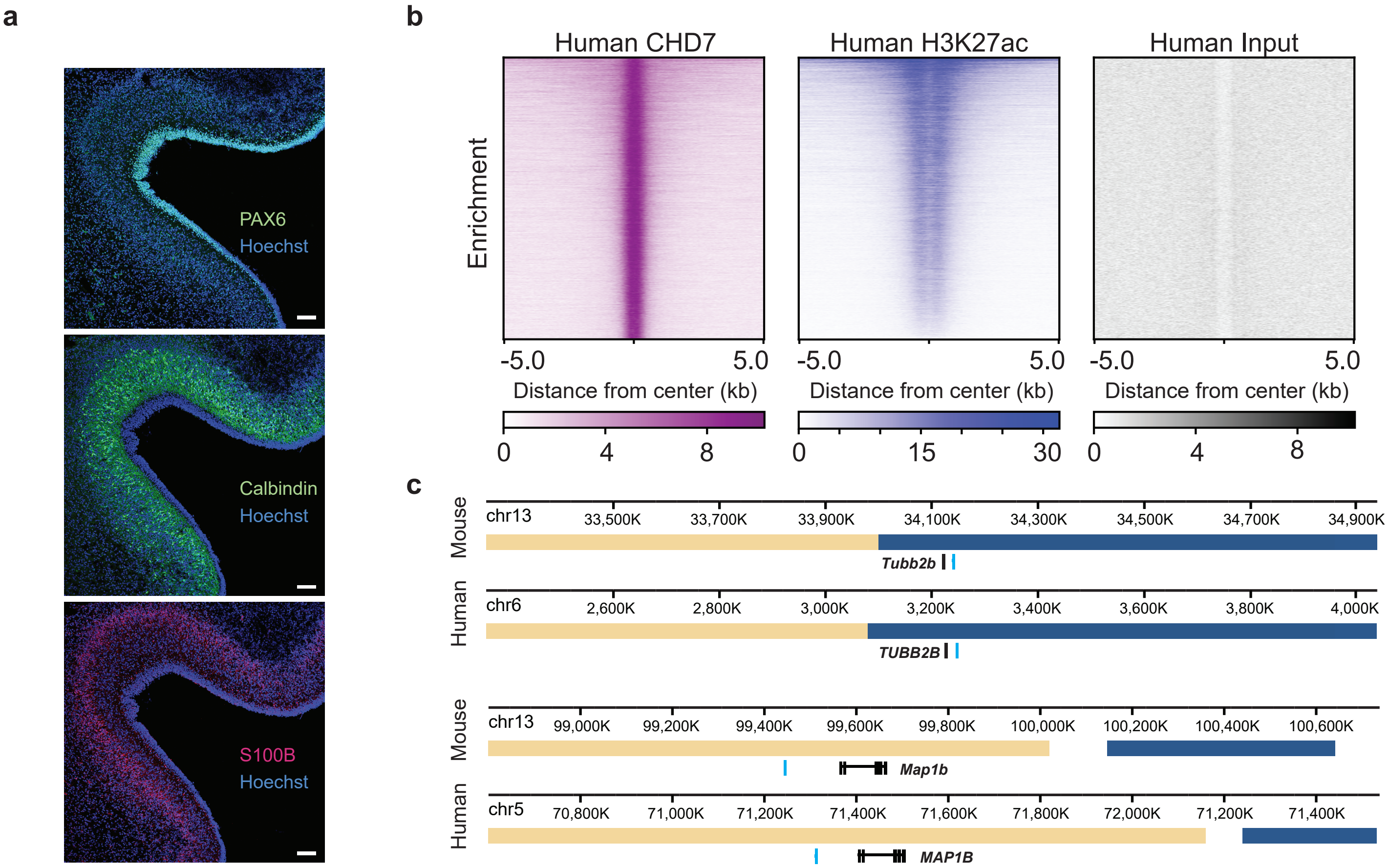
a
PAX6
Hoechst
Calbindin
Hoechst
S100B
Hoechst
b
Human CHD7
Human H3K27ac
Human Input
Enrichment
-5.0
5.0
Distance from center (kb)
0
4
8
15
30
c
Mouse
Human
chr13
33,500K
33,700K
33,900K
34,100K
34,300K
34,500K
34,700K
34,900K
Tubb2b
chr6
2,600K
2,800K
3,000K
3,200K
3,400K
3,600K
3,800K
4,000K
TUBB2B
99,000K
99,200K
99,400K
99,600K
99,800K
100,000K
100,200K
100,400K
100,600K
Map1b
chr5
70,800K
71,000K
71,200K
71,400K
71,600K
71,800K
72,000K
71,200K
71,400K
MAP1B

Supplementary Fig. 6: CHD7 expression and genomic-wide occupancy in granule cell precursors of fetal human cerebellum.

a. Immunohistochemistry analysis of 22 gestational week human cerebellum with Hoechst and antibodies recognizing PAX6 (top), calbindin (middle), and S100B (bottom). Experiment was performed once on the individual human sample. Scale bar: 100um.
b. Heatmap of ChIP-seq signal for human CHD7 (purple), human H3K27ac (blue), and input (black) centered on human CHD7 genomic binding sites (n=17,100).
c. YUE lab Hi-C browser (http://3dgenome.fsm.northwestern.edu/view.php) view of the TUBB2B and MAP1B loci showing the location of conserved CHD7 bound regulatory elements found within conserved topologically associated domains surrounding the TUBB2B and MAP1B loci in mouse cerebellar and in human prefrontal cortical tissue. Mouse data is aligned to mm10 and human data is aligned to hg19. TAD domains are shown in yellow and blue. Enhancers (light blue) and gene loci (black) are shown below TAD domains. Data from Schmitt et al., 2016.

Supplementary Table 1: Number of reads per sequencing sample.

Sample_ID	Total Reads
P4_ChIP_CTCF_cKO_1	30239450
P4_ChIP_CTCF_cKO_2	23882825
P4_ChIP_CTCF_cKO_3	26785055
P4_ChIP_CTCF_Ctrl_1	23887827
P4_ChIP_CTCF_Ctrl_2	22184978
P4_ChIP_CTCF_Ctrl_3	30176045
P4_ChIP_RAD21_cKO_1	25335111
P4_ChIP_RAD21_cKO_2	28519279
P4_ChIP_RAD21_cKO_3	21699710
P4_ChIP_RAD21_Ctrl_1	20240974
P4_ChIP_RAD21_Ctrl_2	22291115
P4_ChIP_RAD21_Ctrl_3	21406366
P4_ChIP_RNAP2_cKO_1	24645904
P4_ChIP_RNAP2_cKO_2	31224374
P4_ChIP_RNAP2_cKO_3	21358159
P4_ChIP_RNAP2_Ctrl_1	25754990
P4_ChIP_RNAP2_Ctrl_2	28502519
P4_ChIP_RNAP2_Ctrl_3	24704851
P4_ChIP_H3K27ac_cKO_1	29513035
P4_ChIP_H3K27ac_cKO_2	22791409
P4_ChIP_H3K27ac_cKO_3	27244501
P4_ChIP_H3K27ac_Ctrl_1	28288347
P4_ChIP_H3K27ac_Ctrl_2	24439089
P4_ChIP_H3K27ac_Ctrl_3	31269223
P4_ChIP_H3K4me1_Ctrl_1	24185291
P4_ChIP_H3K4me1_Ctrl_2	25040047
P4_ChIP_H3K4me1_Ctrl_3	24006539
P4_ChIP_H3K4me1_cKO_2	19872430
P4_ChIP_H3K4me1_cKO_3	25111457
P4_ChIP_H3K4me3_Ctrl	20774101
P4_Input_cKO	24975122
P4_Input_Ctrl	22278616
P4_ChIP_CHD7_Ctrl_1	36321205
P4_ChIP_CHD7_Ctrl_2	34366964
P4_ChIP_CHD7_cKO	60536381
P4_CHD7_Input_cKO	34726055
P4_CHD7_Input_Ctrl	36168280
P4_ATAC_Ctrl_1	124020174
P4_ATAC_Ctrl_2	120750568
P4_ATAC_Ctrl_3	245511484

P4_ATAC_cKO_1	119727870
P4_ATAC_cKO_2	97125142
P4_ATAC_cKO_3	133327052
P4_RNA_Ctrl_1	43762737
P4_RNA_Ctrl_2	44657732
P4_RNA_Ctrl_3	52311021
P4_RNA_Ctrl_4	40845072
P4_RNA_Ctrl_5	44113339
P4_RNA_cKO_1	38058427
P4_RNA_cKO_2	39757228
P4_RNA_cKO_3	41606151
P4_RNA_cKO_4	34955069
P4_RNA_cKO_5	34794895
22w_ChIP_Human_CHD7	46219752
22w_ChIP_Human_H3K27ac	20465108
22w_Human_Input	9049801
Hi-C_Ctrl_1	435398098
Hi-C_Ctrl_2	432510890
Hi-C_Ctrl_3	449314952
Hi-C_cKO_1	417558715
Hi-C_cKO_2	528193699
Hi-C_cKO_3	449493952

Supplementary Table 2: Primers used to genotype mice.

Primer Name	Sequence (5' - 3')
CHD7 floxed allele forward (Genotyping)	TGCAGATGGGACGTTTTCAG
CHD7 floxed allele reverse (Genotyping)	CTGCAAGAACACAGGGCAAG
Control (without Atoh1-Cre transgene) forward (Genotyping)	AAGGTGTGGTTCCAGAAT
Control (without Atoh1-Cre transgene) reverse (Genotyping)	CTCTCCACCAGAAGGCTG
Atoh1-Cre transgene forward (Genotyping)	GATCCGAAAAGAAAACGTTGATG
Atoh1-Cre transgene reverse primer (Genotyping)	TTTTCGTTCTGCCAATATGGATT
