# Supplementary material for: CHARGE syndrome protein CHD7 regulates epigenomic activation of enhancers in granule cell precursors and gyrification of the cerebellum
Source: Nat Commun. 2021 Sep 29;12:5702. doi: 10.1038/s41467-021-25846-3 (PMC8481233; doi:10.1038/s41467-021-25846-3)
Supplement: Supplementary file 2 — Description of Additional Supplementary Files [file 41467_2021_25846_MOESM2_ESM.docx]

**File Name:** Supplementary Data 1
**Description:** Select list of genes used for each gene ontology, Panther pathway, or GREAT analysis performed. Column headers reference the corresponding figures.
