# Supplementary material for: CHARGE syndrome protein CHD7 regulates epigenomic activation of enhancers in granule cell precursors and gyrification of the cerebellum
Source: Nat Commun. 2021 Sep 29;12:5702. doi: 10.1038/s41467-021-25846-3 (PMC8481233; doi:10.1038/s41467-021-25846-3)
Supplement: Supplementary file 3 — Supplementary Data 1 [file 41467_2021_25846_MOESM3_ESM.pdf]

Fig. 1H - CHD7+ P4 Specific	Fig. 1H - CHD7+ P22 Specific			Fig. S1E - CHD7+ P4/P22 Shared			
Abr	Sep5	Esr2	Mpi	Abcd2	Dlg3	Ncam1	Wwc1
Atoh1	Actg1	Fam83g	Mtm1	Abl2	Dlg4	Nck2	Zdhhc13
Cadps2	Adarb1	Fgf14	Ncam1	Adam11	Dnmt3a	Neurl1a	Zic1
Ccnd2	Adcy1	Fos	Negr1	Adcy1	Dock3	Neurod2	Zic4
Cdk5r1	Adcy5	Foxo6	Nlgn1	Adcyap1r1	Dst	Nfasc	Zic5
Cxcr4	Adcyap1r1	Git1	Nos1	Adra2c	En2	Nos1	
Dcx	Adora1	Gnao1	Npas4	Aff1	Epb4.1l3	Npc2	
Dner	Adra1d	Gnas	Nr3c1	Amph	Ercc1	Nrn1	
Egfr	Ank3	Gprc5b	Ntrk2	Ank	Fa2h	Ntrk2	
Gli1	Arntl	Gria2	Park2	Ankrd11	Fez1	Nxph3	
Gli2	Arpp21	Grik2	Pax6	Apba1	Fig4	Pak7	
Kcnj6	Asic1	Grin2a	Pclo	Apba2	Fos	Park2	
Lama1	Atp2b2	Grin2c	Pdyn	App	Fxn	Pbx3	
Lhx1	Baiap2	Grip2	Plcb4	Arntl	Fxyd6	Pde1b	
Neurod1	Bcl11a	Grm1	Ppargc1b	Arpc3	Fyn	Pemt	
Npas3	Cacna1a	Grm4	Prkar1b	Arsb	Gabbr2	Plcb4	
Nrcam	Cacna1c	Grn	Psip1	Asic1	Gal	Plxna2	
Pax6	Cacna1e	Hbegf	Ptpra	Atp1a1	Gpm6b	Pomc	
Plxnb2	Cacna1g	Hcn2	Ptprd	Atp2b2	Hcn1	Ppp1r14c	
Pou3f2	Cacnb4	Hspg2	Rab3b	Bahcc1	Hip1	Prex1	
Pou3f3	Cacng2	Inpp4a	Rarb	Baiap2	Hipk2	Prkcb	
Ptch1	Calb2	Iqsec1	Rasgrf1	Bcr	Idua	Prom1	
Ptk2	Camk2b	Itga5	Reln	Bsn	Inpp4a	Ptch1	
Scn8a	Camk4	Itgb5	Rnd3	Btd	Itga7	Ptpra	
Sema4c	Cck	Jph3	Rptor	Cacna1c	Itgav	Qk	
Thra	Chrna2	Kalrn	Rtn4r	Cacng2	Itm2b	Rbfox2	
Zic4	Chst3	Kcna1	Sez6	Cadps2	Jam3	Reln	
	Cnr1	Kcnb2	Sez6l	Camk2b	Kcna1	Rgs9	
	Col13a1	Kcnip3	Sgcg	Camkk1	Kcna2	Robo3	
	Cplx3	Kcnk3	Sh3gl2	Camkk2	Kcnb2	Rps6ka5	
	Creb1	Kcnk9	Shank2	Cd47	Kcnc1	Scn8a	
	Crhr1	Kdm8	Shh	Cdk5r1	Kcnc3	Sez6	
	Crmp1	Kif13a	Slc17a7	Chd7	Kcnj11	Shank2	
	Cspg5	Kif1a	Slc1a3	Chl1	Kcnj6	Sil1	
	Dact1	Kl	Slc4a3	Chst10	Kif1a	Slc41a3	
	Dclk2	Ksr1	Slc6a1	Clip2	Ky	Slc6a1	
	Disc1	Lep	Slc8a2	Cnr1	Lrpap1	Slc9a1	
	Dll1	Lin7a	Slitrk1	Cntnap1	Lrrn1	Smad2	
	Dnmt3a	Lmna	Snap25	Cplx1	Map2	Snap25	
	Doc2b	Lmx1b	Srgap3	Cplx2	Mapt	Snca	
	Dpp6	Lphn1	Stx1a	Creb1	Mbp	Sptbn4	
	Dpysl4	Lrp1	Stx1b	Csk	Msra	Srgap3	
	Elmod1	Lrp4	Sv2b	Ctnnd2	Myh10	Sstr2	
	En2	Mbp	Syt1	Dab1	Napa	Stxbp1	
	Eps8	Mkks	Syt2	Disc1	Nav2	Synpo	
	Trib2	Vdac1	Thy1	Syt2	Trio	Vac14	
	Trp53	Zdhhc13	Timp2	Timp3	Tuba1a	Vdac1	
	Utrn	Zdhhc17	Tnc	Tnik	Unc13c	Vdac3	
	Vat1l	Zic1	Tnik	Trak1	Utrn	Vldlr	

Fig. S1F - CHD7- P4 Specific

Aatk	Fbln1	Nr2f1	Zeb2
Abcd2	Flnb	Nrcam	Zfp423
Aff1	Foxj1	Nrd1	Zic4
Ago2	Fyn	Otc	Zic5
Akt1	Gad1	Park2	
Als2	Gadd45a	Pax3	
Ank3	Ghrh	Pcdh10	
Apbb1	Gli2	Pdgfra	
Apc	Gpm6b	Phf2	
Atoh1	Gpr37l1	Phgdh	
Atxn2	Gpr6	Plxna3	
Atxn7	Hdac4	Pou3f2	
B4galt1	Id2	Pou3f3	
Bid	Insc	Ppargc1a	
Bmper	Itga6	Ppp1r9b	
Bmpr1a	Itpr1	Prex1	
Boc	Kcnj10	Psmc1	
Ccnd2	Kit	Ptk2	
Cd81	Kl	Ptprs	
Cdk5r1	Lamc1	Qk	
Cdk5rap2	Lats2	Rfx4	
Celsr2	Lhx1	Rgma	
Cep41	Lhx5	Rgs9	
Cga	Lpar1	Rheb	
Chd7	Lrp5	Robo1	
Chl1	Lrp8	Robo2	
Clip2	Macf1	Ror1	
Cntnap1	Map2	Rpl27a	
Cplx2	Map3k4	S1pr1	
Cxcr4	Mdk	Sall1	
D3Bwg0562e	Mkl2	Sema4c	
Dab2ip	Mllt4	Sema6a	
Dclk1	Msx2	Shh	
Dclk2	Mthfd1l	Slc1a2	
Dlx2	Ncoa1	Slc1a3	
Draxin	Ncor2	Slc25a12	
Dyrk1a	Ndp	Slc4a10	
E2f1	Ndst3	Smurf1	
Ebf3	Neurod2	Sox2	
Ednrb	Nfia	Sptan1	
Efnb2	Nfib	Srgap3	
Egfr	Nfix	Sufu	
En1	Nme7	Sumf1	
Erf	Notch1	Syngap1	
Fabp7	Npas3	Tbr1	
Tcf7l1	Tnfrsf21	Vav2	
Tead1	Tsku	Zbtb20	
Tfap2a	Tulp3	Zdhhc17	
Tgif1	Unc5c	Zeb1	

Fig. S1F - CHD7- P4/22 Shared

Abca1	Capn7	Dyrk1a	Kcnq2	Nedd4	Rai1	Ubr4
Actn4	Ccm2	E2f8	Kdm4b	Nedd4l	Rapgef1	Ucp1
Adcy8	Ccnk	Eef1a1	Keap1	Nfasc	Rapgef2	Unc5b
Adora2a	Cd74	Elk3	Kidins220	Nfib	Rara	Unc5c
Adrbk1	Cd81	Ell	Kif1b	Nfic	Rasa3	Usp46
Ago2	Cd8a	En2	Kif3b	Nfix	Rbfox3	Vac14
Agpat3	Cdh2	Epb4.1l3	Klc1	Nlgn3	Reln	Vdac1
Akap6	Cdk5r1	Eps8	Klf7	Notch2	Rgma	Vdac3
Akt1	Cebpe	Erf	Krt10	Nr1d1	Rheb	Wdr19
Akt2	Cecr2	Esrrg	L1cam	Nr2f2	Rims2	Wfs1
Amph	Celf4	Ext1	Lats2	Nrcam	Rnf165	Wnt7b
Ank	Chd2	Fbxw8	Ldha	Nrep	Rnf7	Yap1
Ank3	Chd7	Fdft1	Lin7a	Nrxn2	Satb1	Ybx3
Anp32a	Chd8	Fez1	Lpar5	Nsd1	Satb2	Zbtb20
Anp32b	Chmp4b	Fgf9	Lpp	Nuak1	Scgb1a1	Zdhhc13
Apba2	Chrm4	Foxo1	Lrp1	Pacs2	Scn2a1	Zfand5
Aplp2	Chrnb2	Foxo3	Lrpap1	Pak7	Scn8a	Zfp36l1
App	Cldn11	Fxn	Lrrc4	Park2	Scyl1	Zic1
Arg1	Clip2	Fyn	Ltbp4	Park7	Sec22b	Zic3
Arhgef11	Cnr1	Fzd7	M6pr	Parva	Selplg	Zic4
Arhgef4	Cntfr	Gab1	Macf1	Pax6	Sema3a	Zic5
Arrb1	Cntn1	Gamt	Maml3	Paxip1	Sepn1	Zmiz1
Asxl1	Cog2	Gclc	Map3k1	Pdcd4	Serpinh1	Znrf3
Atf1	Cox4i2	Glce	Map3k4	Pde1b	Setd7	
Atp2a2	Cpe	Gna13	Map4k4	Pde4b	Sez6l	
Atp2b2	Cplx2	Gnai2	Map6	Peg3	Sgms1	
Atp6v0d1	Crtc1	Gnao1	Mbp	Pfdn1	Sh3gl2	
Atp8a1	Ctbp2	Gpm6b	Mcf2l	Pgd	Shank2	
Atpif1	Ctc1	Gse1	Mgll	Phf2	Ski	
Atxn2	Ctnnd1	Hbp1	Mitf	Pik3c3	Slc41a3	
Axin2	Cul1	Hic1	Mllt4	Pisd	Slc7a1	
B9d1	Cux1	Hipk2	Morc3	Pla2g3	Slc8a1	
Bace1	Cyfip1	Hrh2	Mthfd1l	Plcb4	Slc9a1	
Bad	Cyp17a1	Ift122	Mthfr	Plcg1	Smad3	
Baiap2	D430041D05Rik	Ift57	Mtmr2	Pomc	Smo	
Bcar1	Dab1	Igf2r	Mtss1	Ppia	Smurf1	
Bmpr1b	Dact1	Ikzf2	Mxi1	Ppp1r14c	Snap25	
Boc	Dclk2	Inpp4a	Mycbp2	Prkdc	Snca	
Btd	Dctn1	Inpp5a	Myocd	Psap	Sort1	
Cacna1c	Dner	Insig1	Naglu	Ptpn1	Sox5	
Cacna1e	Dnmt3a	Irf2	Nav2	Ptpn11	Sp2	
Cacna1g	Dst	Itsn1	Ncoa3	Ptprj	Sparc	
Cacna2d3	Dtna	Kcnh2	Ncor2	Ptprz1	Spen	
Cacng2	Dync1i2	Kcnj10	Ndfip1	Qk	Sphk1	
Cacng4	Dync1li1	Kcnj11	Ndst1	Rag2	Sptb	
Src	Strap	Tab1	Tmod1	Tph2	Trp53bp2	
Srebf1	Suv420h1	Tapt1	Tom1l2	Traf3ip2	Trp73	
Srgap3	Sv2b	Tbc1d4	Tor1aip1	Trak1	Ttc7	
Stam2	Syt7	Tcf4	Tpcn2	Trio	Ubp1	

Fig. S1F - CHD7- P22 Specific			Fig. S3A - CHD7-Activated		Fig. S3A - CHD7-Repressed			Fig. 4A - Mouse Phenotypes		Fig. 6D - Panther pathways/PMG
Adarb1	Grin2c	Rpl27a	Acsl3	Sema6c	Abhd2	Errfi1	Prkch	Abcd2	Pax6	Actn1
Adcy5	Grn	Rps6ka3	Arhgap44	Slitrk1	Adamts10	Fbln1	Prkcq	Akap12	Ppt1	Arhgap26
Akap5	Gtf2ird1	Rtn4r	Arhgef2	Srrm4	Adamts14	Fgf12	Ptch1	Als2	Ptch1	Asap1
App	Hcn2	Scn8a	Bend6	Ss18l1	Adamts2	Fzd4	Pxn	Atf2	Pten	Col13a1
Arsa	Herc1	Sez6l	Bmi1	Stmn1	Adamts9	Gdf10	Ralb	Atp1b2	Rb1	Col16a1
Astn1	Hipk2	Sh3gl1	Brsk2	Stmn3	Adcy7	Gli1	Rhbdf1	B4galnt1	Rbfox2	Col27a1
Atg5	Idua	Sh3gl2	Casp3	Stmn4	Adcyap1r1	Glp2r	Ror1	Bcr	Rnf2	Col6a1
Atp1b2	Il10	Shank1	Dbn1	Tiam2	Adra2c	Gng7	Rps6ka1	Bmi1	Scn8a	Enah
Avil	Itpr1	Sirt2	Disc1	Tnik	Afap1l2	Gpc1	Rps6ka4	Cacna1g	Sema4g	Evl
Barhl2	Itsn1	Slc25a12	Efna2	Trak2	Amer2	Gpr26	Rxra	Cadps2	Serpine2	Fyn
Bcr	Jph3	Slc2a1	Emb	Tubb2a	Anxa6	Gpr37	S1pr3	Camk4	Slc1a3	Itga5
Cacna1a	Kcna2	Slc7a8	Enah	Tubb2b	Apcdd1	Gucy1b2	Samd14	Cbln3	Sparcl1	Itga6
Cacna1c	Kcnc1	Snap25	Epha7	Ulk2	Aplnr	Icosl	Sema3f	Ccnd1	Sptbn2	Itga9
Cacna2d2	Kcnc3	Snca	Ephb2	Zswim5	Arhgap18	Ifnar2	Sema4a	Cdk5r1	St8sia2	Kif5c
Cacnb4	Kcne1	Syne1	Gpm6a		Arhgap31	Ifngr1	Sema4b	Cdk5r2	Stxbp1	Kras
Cadps2	Kcnj11	Syt2	Gprin1		Arhgef3	Igfbp2	Sema7a	Cers2	Tnik	Lama4
Camk2b	Kcnma1	Syt4	Gprin3		Bambi	Igfbp4	Serpinh1	Cntn1	Trio	Lamb1
Camk4	Kcnq1	Tcap	Gsk3b		Bmp6	Igfbp5	Sesn1	Cp	Tyro3	Limk1
Chd6	Kif1b	Tgm2	H2afz		Cacna1g	Impa2	Sesn2	Ctsl	Unc5c	Map1b
Clip2	Klc1	Trak1	Hist1h1c		Cacng5	Inpp5a	Sgk1	Cxcr4	Wnt5a	Map2k3
Cnr1	Klf9	Trim32	Hist1h1e		Cav2	Ksr1	Sh2b3	Disc1	Zbtb18	Map3k5
Cntn6	Lnpep	Vegfa	Hist1h2ad		Cd93	Lamb1	Shank2	Dpysl5	Zdhhc13	Mapk6
Ctnnd2	Lrp1	Wasf1	Hist1h2af		Cers1	Lfng	Slc27a1	Ebf2		Mapk8
Dbh	Maf	Xpa	Hist1h2ag		Cflar	Ly6h	Smad3	Egfr		Megf9
Dbnl	Mapk10	Zdhhc17	Hist1h2ah		Chrnb4	Lyn	Spry1	En1		Myh14
Dnmt3a	Mapt		Hist1h2ai		Cntfr	Lypd6	Sstr3	Fzd4		Myh7b
Dock3	Mbp		Insm1		Col13a1	Map2k3	St5	Gli1		Myh9
Dscam	Musk		Kif5a		Col15a1	Mertk	Stard13	Glrb		Pak7
Dst	Myh10		Kif5c		Col16a1	Mmp14	Stat5b	Id2		Parva
Dtna	Myo7a		Lgr4		Col18a1	Mmp15	Stk17b	Id3		Parvb
Dtnb	Naglu		Lhx1		Col1a1	Mmp2	Sulf2	Il17rd		Pax6
Ebf2	Neurod2		Lzts1		Col27a1	Nbl1	Syk	Inpp4a		Pfn2
Elavl3	Nfasc		Map1a		Col4a1	Ndrg2	Tle2	Jun		Pik3r3
Elavl4	Nkx6-2		Map1b		Col9a3	Nfat5	Tmem117	Klhl1		Srd5a3
En2	Nlgn3		Map2		Crtap	Nfatc4	Tmem198b	Lama1		Ssh2
Epg5	Nrcam		Mapt		Dact1	Nkd1	Tnfrsf19	Lhx1		Stmn1
Fa2h	Nrd1		Neurod1		Dennd3	Nnat	Trabd2b	Lynx1		Stmn4
Fgf14	Ntrk3		Neurod2		Dkk3	Nos1	Tril	Map1b		Tuba1a
Fgfr1	Nxph3		Neurod4		Dll4	Notch1	Wnt4	Map2		Tubb2a
Frzb	Pik3c3		Nrcam		Dtnbp1	Notch3	Wnt5a	Mapk8ip2		Tubb2b
Fzd5	Plcb4		Ptpro		Dtx1	Nr1d2	Wtip	Mertk		Tubb3
Gabra1	Psap		Rtn4r		Dtx4	Pde1b	Wwtr1	Myo5a		Vcl
Gdf15	Ptprg		Sdccag8		Dusp22	Pde2a		Naglu		
Gnao1	Rasd2		Sema3a		Dusp5	Pecam1		Neurod1		
Gnaq	Rbfox1		Sema3d		Dusp6	Pgf		Neurod2		
Gnb5	Rbfox2		Sema3e		Efnb3	Plce1		Neurod4		
Gria2	Rnf103		Sema3g		Ephb6	Plxna1		Nfasc		
Grin1	Robo3		Sema4f		Eps8l3	Plxnd1		Nrcam		
Grin2a	Rora		Sema4g		Erlin1	Ppp1r1a		Pafah1b1		
